# The SOS Response Master Regulator LexA Is Associated with Sporulation, Motility and Biofilm Formation in *Clostridium difficile*

**DOI:** 10.1371/journal.pone.0144763

**Published:** 2015-12-18

**Authors:** Beata M. Walter, Stephen T. Cartman, Nigel P. Minton, Matej Butala, Maja Rupnik

**Affiliations:** 1 National Laboratory for Health, Environment and Food, NLZOH, Maribor, Slovenia; 2 The Clostridia Research Group, BBSRC/EPSRC Synthetic Biology Research Centre, School of Life Sciences, Centre for Biomolecular Sciences, University of Nottingham, Nottingham, NG7 2RD, United Kingdom; 3 Biotechnical Faculty, University of Ljubljana, Department of Biology, Ljubljana, Slovenia; 4 Faculty of Medicine, University of Maribor, Maribor, Slovenia; 5 Centre of Excellence for Integrated Approaches in Chemistry and Biology of Proteins, Ljubljana, Slovenia; Universidad Andres Bello, CHILE

## Abstract

The LexA regulated SOS network is a bacterial response to DNA damage of metabolic or environmental origin. In *Clostridium difficile*, a nosocomial pathogen causing a range of intestinal diseases, the *in-silico* deduced LexA network included the core SOS genes involved in the DNA repair and genes involved in various other biological functions that vary among different ribotypes. Here we describe the construction and characterization of a *lexA* ClosTron mutant in *C*. *difficile* R20291 strain. The mutation of *lexA* caused inhibition of cell division resulting in a filamentous phenotype. The *lexA* mutant also showed decreased sporulation, a reduction in swimming motility, greater sensitivity to metronidazole, and increased biofilm formation. Changes in the regulation of toxin A, but not toxin B, were observed in the *lexA* mutant in the presence of sub-inhibitory concentrations of levofloxacin. *C*. *difficile* LexA is, therefore, not only a regulator of DNA damage but also controls many biological functions associated with virulence.

## Introduction

The SOS regulatory network is a part of bacterial response to DNA damage [1]. The master regulators of this system are the global transcriptional repressor LexA and an inducer, the recombinase protein RecA [2,3]. The mechanism of LexA activation is best studied in *Escherichia coli*. In clostridia, a LexA/RecA based SOS network was reported in *Clostridium perfringens* where it was induced by methyl methane sulfonate (MMS) and UV radiation [4]. Moreover, LexA binding sites have been identified in both *C*. *perfringens* and *Clostridium acetobutylicum* where addition of an operator sequence was shown to increase responsiveness to irradiation [5].

Besides the repression of DNA damage responsive genes, LexA also controls several other genes including virulence factors. For instance, LexA was reported to regulate in *Vibrio cholerae* the production of temperate phage CTX, encoding cholera toxin [6] and colicins in *E*. *coli* [7,8]. Chellappa and co-workers [9] showed that induction of the SOS response represses flagellar motility but increases biofilm formation in *Pseudomonas aeruginosa*. Furthermore, the resistance of *Bacillus subtilis* spores to DNA double-strand breaks has been correlated to RecA and its accessory proteins [10].

*C*. *difficile* is a nosocomial, Gram-positive, anaerobic pathogen which causes a range of intestinal diseases associated with antibiotic treatment [11]. An *in-silico* study coupled to an *in-vitro* LexA-DNA interaction analysis by surface plasmon resonance spectroscopy, identified 16 LexA target sites within genomes of *C*. *difficile* [12]. The identified putative LexA operators were found within the pathogenicity locus PaLoc, which encodes two large clostridial toxins TcdA and TcdB, and in the promoter region of the *sspB* gene that encodes for a protein involved in sporulation [12]. Interestingly, minor changes in levels of some SOS proteins (RecA, LexA, UvrB, UvrC) were observed in a Spo0A deficient *C*. *difficile* mutant [13]. Moreover, recent analysis of a stable metronidazole-resistant *C*. *difficile* isolate suggested a possible role for DNA repair genes (*uvrABC* operon) in the mechanism of resistance to this drug [14]. LexA binding sites were found in promoter regions of the house-keeping genes *rplR* and *rpoB*, suggesting the network controls various biological functions including pathogenicity related properties [12].

To further analyse the possible association of LexA with *C*. *difficile* virulence we have constructed a *lexA* mutant that displays a constitutively induced SOS response and studied several phenotypic properties.

## Materials and Methods

### Strains and plasmids used in this study

ClosTron mutagenesis was performed in *C*. *difficile* R20291 (BI/NAP1/027). Phenotypic analysis of the *lexA* mutant was compared to the R20291 wild-type strain and *C*. *difficile* 630 (012). The list of strains used in this study, including *E*. *coli* strains and plasmids applied in mutagenesis, is shown in S1 Table.

#### CdiR20291-lexA238a::CT strain construction and complementation

ClosTron-mediated mutagenesis of *lexA* in *C*. *difficile* R20291 was performed as previously described [15]. The outputs of the intron targeting and design tool from www.clostron.com for the insertion site at 238|239 nucleotide (antisense) are shown in S1 File. Construction of re-targeted pMTL007C-E2 vector plasmids was outsourced (DNA2.0, USA; Heap, Kuehne [15]). *LexA* mutant clones were PCR screened with screening primers and EBS universal primer (primer sequences shown in S2 Table). Insertion of the intron was verified by sequencing PCR amplified *lexA*. Single insertion was verified by Southern Blot. Chromosomal DNA for Southern blot was extracted using high quality phenol chloroform method as described elsewhere [16]. Southern blots were performed with DIG High Prime DNA Labelling and Detection Starter Kit I (11 745 832 910, Roche) according to the manufacturer’s protocol. The CdiR20291-lexA238a::CT strain called here: *lexA* mutant was complemented with an expression plasmid pMTL84151 (GenBank: FJ797649.1) carrying *lexA* gene constructed through the use of Splice Overlap Extension (SOE) PCR [17]. The *lexA* gene was amplified from R20291 genomic DNA using primers containing *Nde*I and *Bam*HI restriction sites (S2 Table) and cloned into plasmid vector pMTL84151. Transfer of the vector pMTL84151::lexA into *C*. *difficile lexA* mutant was accomplished by conjugation with the *E*.*coli* CA434 donor as described for ClosTron delivery [18] (S1 Table). The CdiR20291-lexA238a::CT::pMTL84151::lexA strain is called here complemented *lexA* mutant.

#### Routine growth of *C*. *difficile* strains

Prior to each experiment, fresh cultures were restreaked from a -80°C stock, in an anaerobic workstation (Don Whitley Scientific, United Kingdom) at 37°C and incubated for 24–48 h. The wild-type strains were cultured in BHIS cc (brain heart infusion supplemented with 0.1% (w/v) L-cysteine (Sigma-Aldrich) and 5 mg/ml yeast extract (Oxoid) and *Clostridium difficile* supplement (cc) (250 μg/ml D-cycloserine and 8 μg/ml cefoxitin (Sigma-Aldrich)). The *lexA* mutant strain was grown on BHISLm (BHIS cc supplemented with 20 μg/ml lincomycin). The plasmid complemented *lexA* mutant strain was cultivated on BHISTm (BHIS cc supplemented with 15 μg/ml thiamphenicol) to prevent loss of complementation plasmid carrying thiamphenicol resistance gene.

### Antibiotic susceptibility

Antibiotic susceptibility testing was performed using Etest^®^ (AB bioMeriux) as described earlier [19] for the following antibiotics: erythromycin (EM); tetracycline (TC); doxycycline (DC); clindamycin (CM); amoxicillin (AC); rifampicin (RI); metronidazole (MZ); vancomycin (VA); piperacyllin/tazobactam (PTc), and; levofloxacin (LE). The MIC assay for ampicillin (AM) was performed using the microbroth dilution assay in an anaerobic cabinet using pre-reduced BHIS media. Suspension of a fresh culture at OD_620_ 0.6 was prepared and inoculated at ratio 1:100 in 96-well plate into media containing two-fold dilutions of antibiotic (32 to 0.125 μg/ml) and incubated for 24–48 h. The MIC was determined by measurement of turbidity with Sunrise^™^ (Tecan) at OD_620_. MIC was determined in triplicate and in three independent experiments. The MIC breakpoint was specified where possible [20, 21, 22, 23].

### Cell morphology and growth curve analysis

Cell morphology was examined under light microscopy. The strains were revived from a stock, restreaked onto appropriate medium and incubated overnight. Small samples were harvested in triplicate and slides were Gram stained with PREVI-color-gram (BioMerieux).

Growth curve analysis was performed in four different liquid media: BHIS (described above); BHISG (BHIS supplemented with 1% (w/v) glucose); PY (1% (w/v) bacto peptone, 1% (w/v) yeast extract, salts: 8 μg/ml CaCl_2_, 8 μg/ml MgSO_4_, 40 μg/ml K_2_HPO_4_, 40 μg/ml KH_2_PO_4_, 0.4 mg/ml NaHCO_3_, 80 μg/ml NaCl) and PYG (PY supplemented with 1% (w/v) glucose).

In all growth curve experiments, a 0.3 ml aliquot of overnight culture was diluted in 5 ml of liquid medium and incubated for 5 h. The growth curve was set up by inoculation of fresh medium with the 5 h old culture in a 1: 1000 ratio. The optical density was checked every 2 h for the first 16 h then at 26, 34 and 50 h. Additional samples for microscopical analysis and cytotoxicity assay were also taken at each time point. The experiment was performed in at least 3 independent replicas.

### Extraction and purification of the *C*. *difficile* total RNA

Total RNA of wild-type and *lexA* mutant was extracted using RNAprotect Bacteria Reagent (76506, QIAGEN) QIAzol lysis reagent (79306, QIAGEN) combined with RNeasy Mini Kit (74104, QIAGEN). RNAprotect Bacteria Reagent in a 1:1 ratio was added to the sample taken at 12 and 24 h during bacterial growth, incubated for 5 min at room temperature and centrifuged for 10 min at 5000 x g. The supernatant was removed and pellet stored at -80°C for up to 5 days. Thereafter, pellets were thawed, suspended in 2 ml of ethanol:methanol mixture [1:1] and incubated at -80°C for minimum 20 min. After centrifugation for 10 min at 3000 rpm at 4°C the pellet was washed twice in 500 μl of TE buffer (10 mM Tris, 1 mM EDTA, pH 7.6) and once in 200 μl of SET buffer (50 mM NaCl; 5 mM EDTA; 30 mM Tris-HCl pH 7.0; 10% SDS (w/v)). The primary lysis was undertaken in 83 μl of TL buffer (50mM Tris, pH 6.5; 50 mg/ml lysozyme), 2 μl of SUPERase In^®^ RNase inhibitor (AM2694, Life technologies) and 10 μl proteinase K mix (19131, QIAGEN) for 45 min at 37°C with mixing 350 rpm. The secondary lysis was performed in 600 μl of QIAzol lysis for 5 min at room temperature. Nucleic acids were separated by adding 140 μl of chloroform and incubated 2–3 min followed by 15 min centrifugation at 13200 rpm (maximal speed) at 4°C. The upper layer was mixed with 1.5 x volume of 100% ethanol. The sample, including any precipitate was transferred into RNeasy Mini Spin column (74104, QIAGEN) and centrifuged for 15 s at 8000 x g. Further RNA purification with RNeasy Mini Kit (74104, QIAGEN) was performed according to manufacturer description. The concentration of isolated RNA was measured with a NanoDrop ND-1000 spectrophotometer, adjusted to 100 ng/μl and stored at -80°C.

### RecA gene expression measurement—reverse transcription quantitative real time PCR

Reverse transcription quantitative real time PCR (RT-qPCR) was performed on Rotor-Gene Q (QIAGEN) with rotor Gene Probe RT-PCR kit (204574, QIAGEN) according to the manufacturer instructions. Briefly, 12.5 μl of 2x Rotor-Gene Probe RT-PCR Master Mix (containing HotStarTaq Plus DNA polymerase, Rotor-Gene Probe RT-PCR Buffer and dNTPs mix); 0.25 μl of Rotor-Gene RT Mix (containing Omniscript^®^ and Sensiscript^®^ Reverse Transcriptases); 1.2 μl of each *recA* and *rpsJ* primers; 0.6 μl of each *recA* and *rpsJ* probe and 4.75 μl per 1.5 μl sample (150 ng/reaction) were used. Reverse transcription was performed at 50°C for 30 min followed by PCR initial activation step at 95°C for 5 min and 40 cycles of 3 s denaturation step at 95°C and 12 s annealing/extension step at 59°C. Cycling was acquired on green (for *rpsJ*) and orange (for *recA*) channels. Expression levels of *recA* were tested in BHIS and BHISL (BHIS supplemented with 8 μg/ml LE) at 12 h and 24 h. The primers and the probe for *recA* amplification were selected with Primer3 (http://simgene.com/Primer3). The *rpsJ* gene was selected for normalisation (reference gene) after optimisation. Modified primers proposed by Metcalf *et al*. [24] for the *rpsJ* gene were used. The probe for *rpsJ* was designed with Primer3. All oligonucleotides are listed in S2 Table. The experiments were performed in two biological replicates (RNA originating from two independent culture extractions) and two technical replicates (biological sample tested in triplicates in two independent PCR amplifications). Ct values were analysed using a relative expression ratio with an equation: $2^{-1\Delta \Delta C_{{}_{T}}}$ as applied previously [25, 26]. The expression ratio was calculated for the *lexA* mutant, *lexA* mutant grown in the presence of LE and the R20291 wild-type grown in the presence of LE relative to the R20291 wild-type calibrator.

### Sporulation

Sporulation assays for the wild-type and *lexA* mutant strains were performed by enumeration of total and heat resistant CFU (colony forming units) for up to 6 days with sampling at 24 h intervals. Cultures were cultivated in BHIS and PY medium as described for the growth curve experiments and the assay was performed as described by Burns and co-workers [27] with the difference that the heat treatment was performed at 70°C for 20 min. The spore number was defined as CFU following heat treatment. Sporulation frequencies were determined as the ratio of the number of heat resistant CFU and the total number of CFU before heat treatment. At least three independent experiments were performed and standard errors of the means were calculated.

### Biofilm formation

The biofilm formation assay was performed for the *C*. *difficile* R20291 wild-type, *lexA* mutant, plasmid complemented *lexA* mutant and strain 630 as described by Dapa and co-workers [28]. The biofilm was generated in a tissue culture treated 24-well polystyrene plate (CLS3527, Sigma-Aldrich). The optical density OD_570_ of crystal violet retained by the biofilm was measured on a Sunrise^™^ instrument (Tecan). Non-inoculated broth medium was used as a negative control. The average OD_570_ of biofilm cell mass and standard deviation was calculated based on three independent experiments performed in duplicate.

### Motility test

To assess the motility of the wild-type strain and *lexA* mutant, the following protocol was adopted. A 0.3 ml aliquot of an overnight culture was diluted into 5 ml of liquid medium, incubated for 5 h, further diluted in 1:1000 ratio, incubated for 16 h and the turbidity adjusted to the OD_620_ value of 0.6. Motility was measured by either stabbing cell suspensions into solidified media in a test tube, or by their inoculation onto the surface of agar media in Petri dishes. In the former case, a sterile toothpick carrying culture suspension was stabbed, in triplicate, into a test tube containing 5 ml of pre-reduced 0.5% (w/v) BHIS agar. For the latter test, 10 μl drops of a culture were spotted onto the surface of the same media in Petri dishes. In both cases incubation was for 2 days at 37°C, in an anaerobic workstation and two independent experiments were performed.

### Cytotoxicity assay

Vero cells (ATTC) were routinely cultured in 75 cm^2^ Flasks in Dulbecco`s Modified Eagle Medium (DMEM) supplemented with 10% (v/v) Foetal bovine serum (FBS), 1% (v/v) sodium pyruvate, 1% (v/v) MEM non-essential amino acids solution, 100X, 1% (v/v) penicillin/streptomycin at 37°C with 5% (v/v) CO_2_. Cytotoxic assays were performed in 96 well plates on 80–90% confluent Vero cells in DMEM supplemented with a lower concentration of FBS (1% (v/v)).

*C*. *difficile* samples taken at different time points during bacterial growth were centrifuged 5 times at 13200 rpm for 10 min and the supernatant was stored at -20°C for time period not exceeding 7 days prior to intoxication. The five-fold dilutions of supernatants were prepared and applied to cells in 1:10 (v/v) ratio and incubated overnight. Sterile media were used as a negative control. The serial dilution titre that exhibited 50% cytotoxicity was recorded and plotted on a logarithmic scale. The experiment was performed in 3 independent replicates.

### ELISA for toxin A and toxin B

ELISA for the separate detection of *C*. *difficile* Toxin A or Toxin B in suspensions (TGC-E002-1, tgcBIOMICS) was used to detect and quantify the level of both toxins being produced by the wild-type and *lexA* mutant strains. Four different time points were tested (4, 12, 26 and 50 h) during growth in PY medium and PY medium supplemented with 8 μg/ml LE. The samples were taken throughout the growth curve, prepared according to the manufacturer’s instructions and a dilution of 1:10 prepared in the dilution buffer provided in the kit. The ELISA assay was performed according to manufacturer`s description. The experiment was performed in 2 independent replicates.

## Results and Discussion

### Mutagenesis of the *lexA* gene in *C*. *difficile* R20291

**Fig 1:**
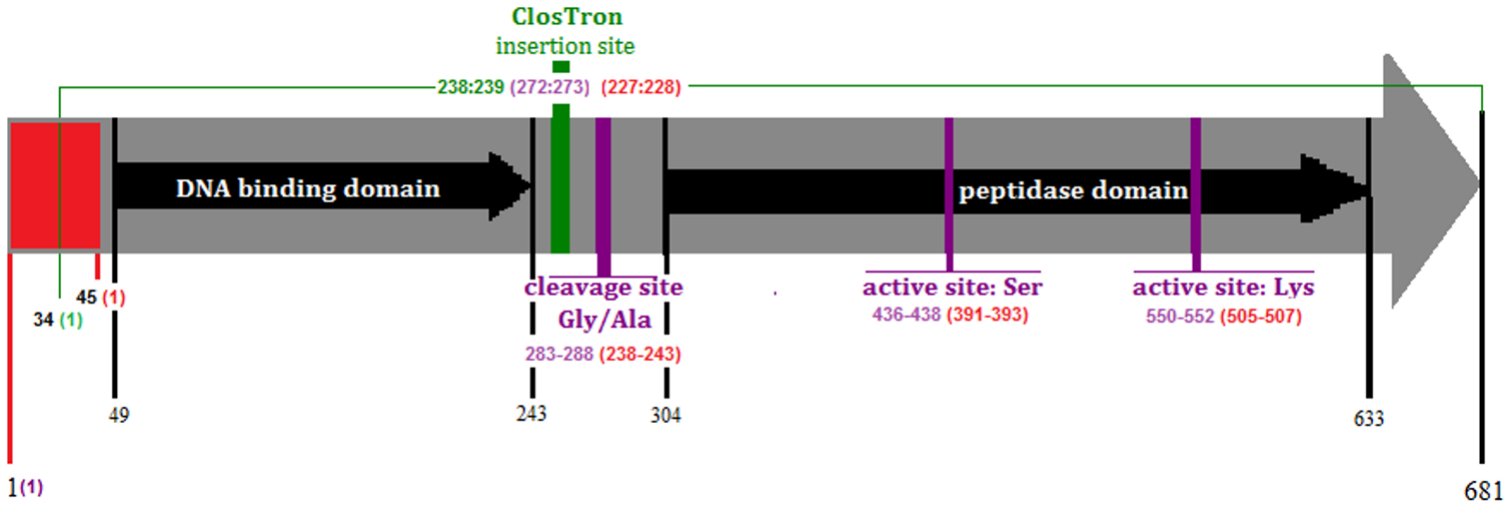
Representation of the *C*. *difficile* R20291 *lexA* gene. The arrow denotes the nucleotide sequence of the R20291 *lexA* gene. The fragment marked in red shows a 45 nucleotide sequence which is absent in the genome of *C*. *difficile* 630. Small black arrows indicate the position of sequences coding for LexA domains. The position of the cleavage site and catalytic residues is marked in purple. The ClosTron insertion site is marked in green. The green bracket beginning at nucleotide 34 indicates the sequence used for ClosTron design. The numbers of R20291 *lexA* nucleotides are presented below the arrow in black. The numbers in purple indicate the nucleotide numbering of R20291 active/insertion sites which correlates with numbers in black. The numbers in green indicate the nucleotide numbers of a sequence used for ClosTron design. The numbers in red indicate the nucleotide numbering of CD630 active/insertion sites.

To study the characteristics of the SOS response in *C*. *difficile* we attempted to make two fundamentally different classes of mutants. In one instance a knock-out mutant made by insertional inactivation that was no longer able to produce LexA protein, and in the other case a mutant encoding a variant, non-cleavable LexA in which the Ser residue at position 135 was replaced with Ala. The methodology adopted to make the latter mutant was the allelic exchange method described by Cartman and co-workers [29]. However, in no instance was this successful either in strain 630 or R20291. The most likely explanation for this failure is that mutation leading to formation of non-cleavable LexA is lethal for *C*. *difficile* as the protein seems to repress several essential genes [12]. We were, however, able to obtain an insertional ClosTron mutant in strain R20291 which would result in the production of a dysfunctional LexA protein (designation: CdiR20291-lexA238a::CT called here: *lexA* mutant) (Fig 1). The *lexA* deletion is lethal for *E*. *coli* unless another gene, *sulA*, is inactivated. The SulA protein arrests cell division after DNA damage [30]. In contrast, *lexA* deficient *B*. *subtilis* and *B*. *megaterium* strains are viable [31, 32]. In the *C*. *difficile lexA* mutant, the ClosTron intron is inserted between a region of DNA encoding the LexA DNA binding domain sequence and a region of the gene coding for the LexA dimerisation and catalytic domain (Fig 1). Therefore, the mutant strain could synthesize repressor residues 1–92 harbouring the DNA binding domain fused to the several intron-encoded amino acids. Thus, the LexA-derivative might retain some affinity for SOS target sequences. To assist the characterisation of the mutant, a complemented strain was constructed. Complementation was achieved by cloning the wild-type *lexA* gene into the expression plasmid pMTL84151 (pMTL84151::lexA) which was introduced into the *lexA* mutant strain to yield the strain CdiR20291-lexA238a::CT::pMTL84151::lexA, called here complemented *lexA* mutant strain.

### Morphological and growth properties of *C*. *difficile lexA* mutant

**Fig 2:**
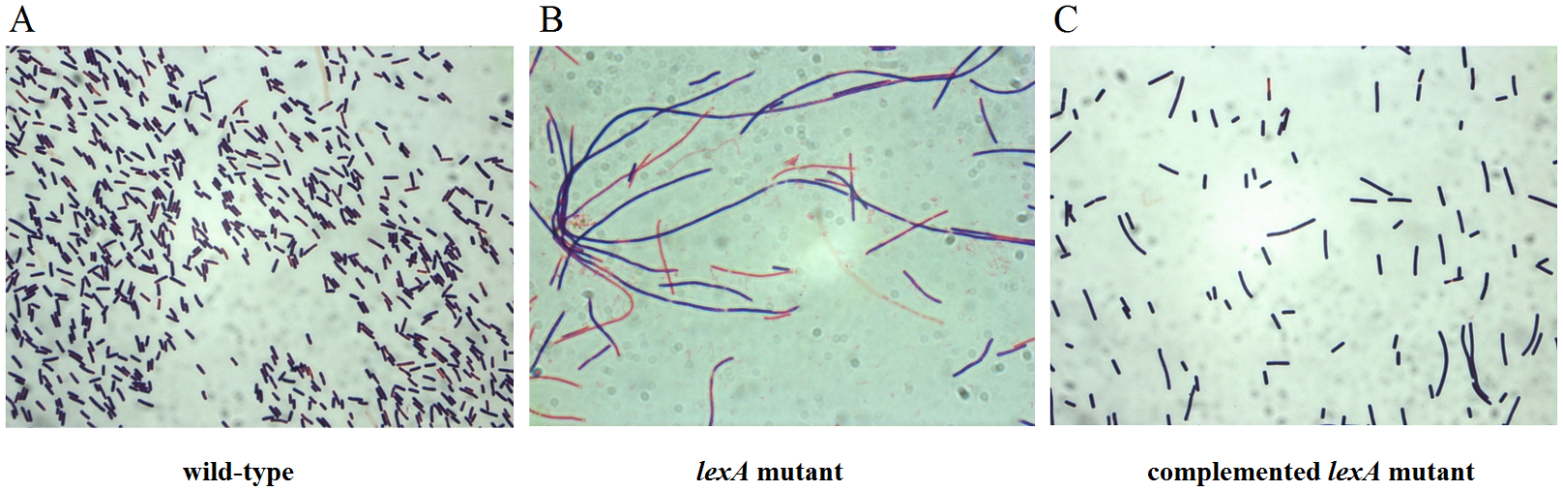
Cell morphology after Gram staining. (A) R20291 wild-type; (B) *lexA* mutant (CdiR20291-lexA238a::CT); (C) plasmid complemented *lexA* mutant (CdiR20291-lexA238a::CT::pMTL84151::lexA).

The characteristics of the *lexA* mutant were compared to its isogenic parent, *C*. *difficile* R20291, *C*. *difficile* 630 (in biofilm formation experiments only) and to the complemented *lexA* mutant strain. The initial examination under light microscope indicated that the *lexA* mutation altered cell morphology, causing them to grow as abnormal, filamentous rods (Fig 2B). This abnormality was alleviated in the complemented strain where the morphology of growing cells was indistinguishable from that of the wild-type parent strain (Fig 2). A filamentous phenotype is indicative of a defect in cell division inhibition and has previously been described to be associated with the activated SOS response [31, 32].

**Fig 3:**
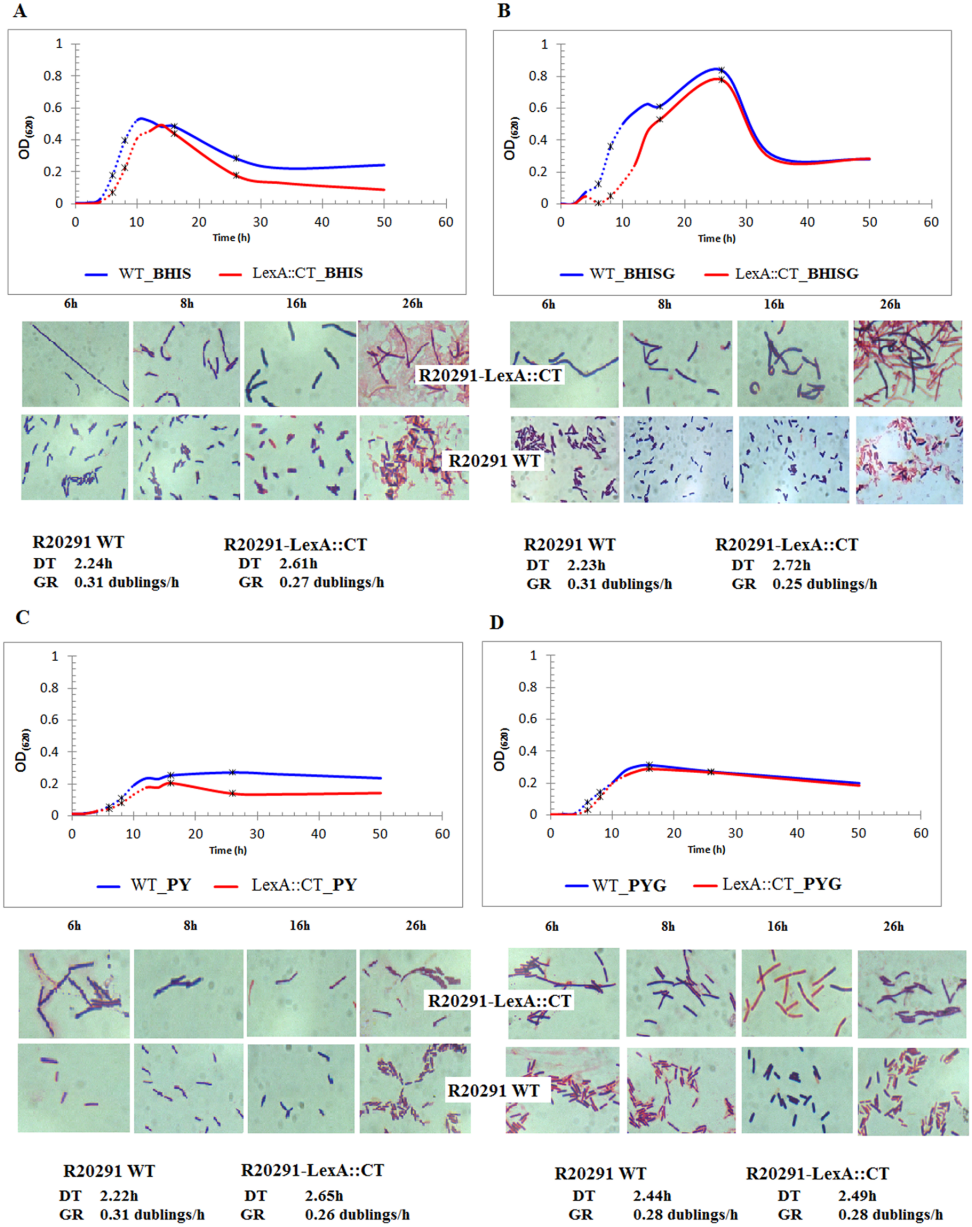
Growth and morphology of the R20291 wild-type (WT) and the *lexA* mutant (LexA::CT) in various media. Growth and cell morphology in (A) BHIS medium; (B) BHISG medium; (C) PY medium (D) PYG medium. Growth was analysed by measurement of turbidity at OD_(620)_ at time 0, 2, 4, 6, 8, 10, 12, 14, 16, 26, 34 and 50 h. The dotted curve shows data taken for calculation of DT—doubling time and GR—growth rate. The time points of sampling for Gram staining (6, 8, 16 and 26 h) are shown on curves with black asterisks.

A filamentous phenotype of the *lexA* mutant was observed in all four media tested (BHIS, BHISG, PY, PYG) (Fig 3; S2 File). The wild-type and the *lexA* mutant growth rate and doubling times, as computed with online calculator [33], were comparable between the two strains (Fig 3). The *lexA* mutant strain showed a comparable growth curve to the wild-type in BHIS and PY media unless glucose was added.

### SOS response is constitutively induced in *lexA* mutant

The lack of a functionally active LexA protein in the mutant strain may lead to the constitutive expression of the SOS response. To test this hypothesis we followed the expression of the core SOS gene, *recA* in the *lexA* mutant and in the wild-type parent strain when grown in BHIS medium.

**Fig 4:**
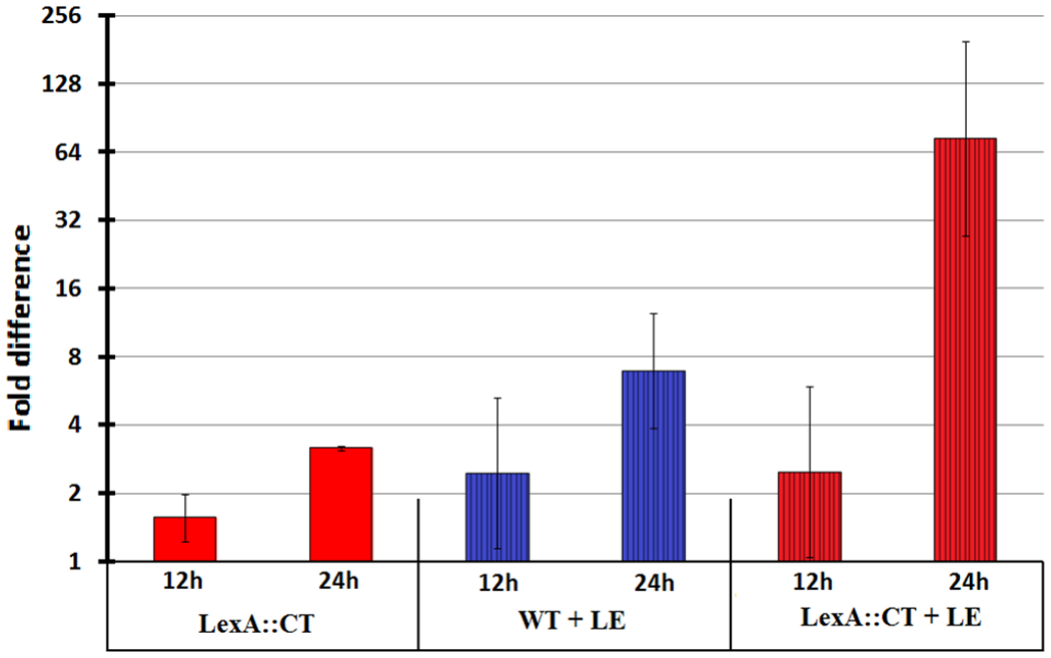
Fold differences in *recA* gene expression at 12 h and 24 h of growth. The columns represent the fold difference in *recA* expression of the test sample relative to the R20291 wild-type calibrator. Red colour designates the *lexA* mutant (LexA::CT), while the blue colour designates the R20291 wild-type (WT). The stripped columns represent those strains grown in the presence of levofloxacin (8 μg/ml). The plain red columns show the fold change in *recA* expression of the *lexA* mutant relative to the calibrator. The striped blue columns show the fold change in *recA* expression of R20291 grown in levofloxacin (8 μl/ml) relative to the calibrator. The stripped red columns show the fold change in *recA* expression of *lexA* mutant grown in levofloxacin (8 μl/ml) relative to the calibrator. Low relative expression ratio expressed as a fold difference indicates similar gene expression by the test sample and the calibrator. High relative expression ratio indicates higher fold of expression by the tested strain relative to the calibrator.

In the *lexA* mutant, a 1.6-fold change in *recA* expression was observed after 12 h of growth (compared to the wild-type calibrator) and a 3.2-fold change after 24 h (S3 Table, sample 1; Fig 4), thus data imply that LexA-regulon genes are constitutively expressed in the mutant.

In addition, we followed *recA* expression in the *lexA* mutant and in the R20291 wild-type in the presence of 8 μg/ml LE, an antibiotic known to induce the SOS response in *E*. *coli* and *S*. *aureus* [34, 35]. The wild-type and its *lexA* mutant exhibited LE resistance with an MIC of greater than 32 μg/ml (S4 Table), which is related to amino acid substitution Thr82-Ile in GyrA [36]. Thus, despite the fact that in *V*. *cholerae* and *E*. *coli* fluoroquinolones were shown to induce the SOS response at concentrations that were 100- to 230-fold lower than the MIC [37, 38, 39], we used a relatively high concentration of LE for our experiments (8 μg/ml). The effect of LE on *recA* expression was more significant in the strain lacking the functional LexA (mutant) than in its isogenic strain (Fig 4, S3 Table). In comparison to the non-induced growth of the wild-type, we observed a 2.5-fold increase in *recA* mRNA levels after 12 h of growth in the LE induced *lexA* mutant and its isogenic parent strain. Furthermore, for the 24 h time point we observed a 6.9-fold increase and a 73.5-fold increase in *recA* expression in the wild-type and in the *lexA* mutant strain, respectively. Therefore, our results suggest that *recA* expression is not solely LexA-controlled but is also regulated by a LexA independent mechanism that is sensitive to LE.

### Decreased sporulation of LexA mutant

**Fig 5:**
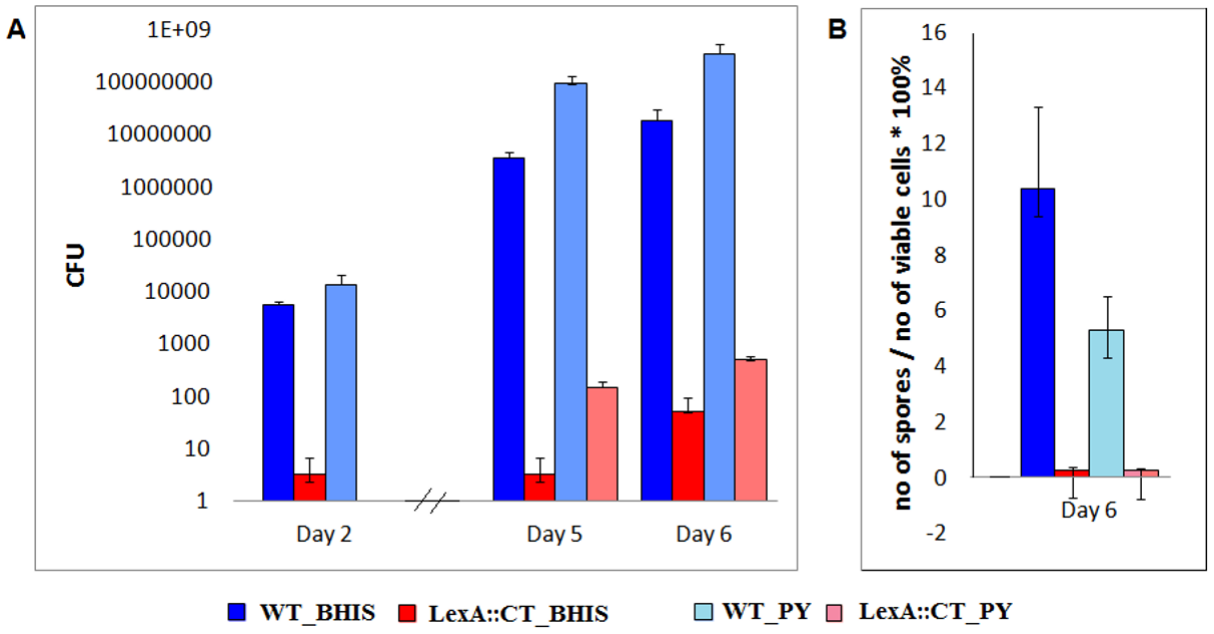
Sporulation properties of *lexA* mutant compared to its parent R20291 strain. (A) The colony formation following heat treatment (70°C, 20 min) of *C*. *difficile* strain R20291 (WT) and the *lexA* mutant (LexA::CT) in BHIS and PY medium. (B) The sporulation frequency is based on the ratio of heat resistant spores and total colony forming units of *C*. *difficile* R20291 wild-type and the *lexA* mutant. The cultures were grown in BHIS and PY and plated on BHIS Tc. The bars stand for averages of three independent experiments and error bars indicate standard errors of the means. The detection limit for colony counts was 50 CFU/ml for the wild-type and 10 CFU/ml for the mutant.

R20291 produces lower number of spores in comparison to strain 630. Previous studies have shown that cultures of R20291 contain 1x10^5^ -1x10^6^ heat resistant CFU/ml after 5 days of incubation, while *C*. *difficile* 630 produces 1x10^8^ heat resistant CFU/ml after the same time period [27, 40]. We observed, 3.5x10^6^ heat resistant CFU/ml for the R20291 wild-type strain after 5 days which remains in agreement with these studies [27, 40] (Fig 5).

In the *lexA* mutant significantly less spores were detected in comparison to the wild-type (Fig 5). After 6 days of growth in BHIS medium, 1.8x10^7^/ml spores of the wild-type R20291 strain were produced, while approximately 5 log_10_ less spores were detected in the *lexA* mutant (1.4x10^2^ CFU/ml). In less nutritious PY medium, the spore number of the R20291 wild-type strain increased ~1 log_10_ relative to BHIS (3.5x10^8^ CFU/ml) while there was an approximately 3.6-fold increase in spore numbers in the *lexA* mutant (5.0x10^2^CFU/ml) (Fig 5), yet still significantly less than the wild-type. The sporulation frequency of R20291 was 10%, while the sporulation frequency of the *lexA* mutant was approximately 0.2% (Fig 5B).

The decrease in sporulation exhibited by the *lexA* mutant indicates that the intact SOS system plays a role in *C*. *difficile* sporulation, a phenomena that was also observed in *B*. *subtilis*. The LexA regulon of *B*. *subtilis* includes *cwlD*, which encodes a cell-wall hydrolase which is necessary for spore formation and dormancy [41, 42]. In this bacterium, LexA also represses the expression of the *sda* gene which encodes for the checkpoint factor that prevents sporulation in response to DNA damage [43]. Although Sda is absent in *C*. *difficile* [44], and therefore cannot be related to decreased sporulation, we previously reported that LexA interacted *in vitro* with the putative promoter region of another gene involved in sporulation, *sspB* [12]. Thus, the LexA network of *C*. *difficile*, in common with *B*. *subtilis*, also controls sporulation. However, our data implies that regulation occurs via a different pathway which might involve *sspB*. Moreover, Pettit and co-workers [13] observed changes in expression of SOS related proteins between *C*. *difficile* wild-type and a *C*. *difficile spo0A* deficient mutant (log2 difference: *rec*A (-0.14), *lex*A (0.18), *uvr*B (0.18), *uvr*C (0.34)). Thus, several SOS and sporulation genes seem to be co-dependent on LexA and Spo0A. The SOS response is activated upon DNA damage while the spores are produced as a bacterial response to overcome and survive harsh environmental conditions. Decreased sporulation upon induction of the SOS response could be beneficial for the bacterium as it ensures the cell does not enter sporulation before the repair of damaged DNA.

### Changed motility and higher biofilm formation by the *lexA* mutant

**Fig 6:**
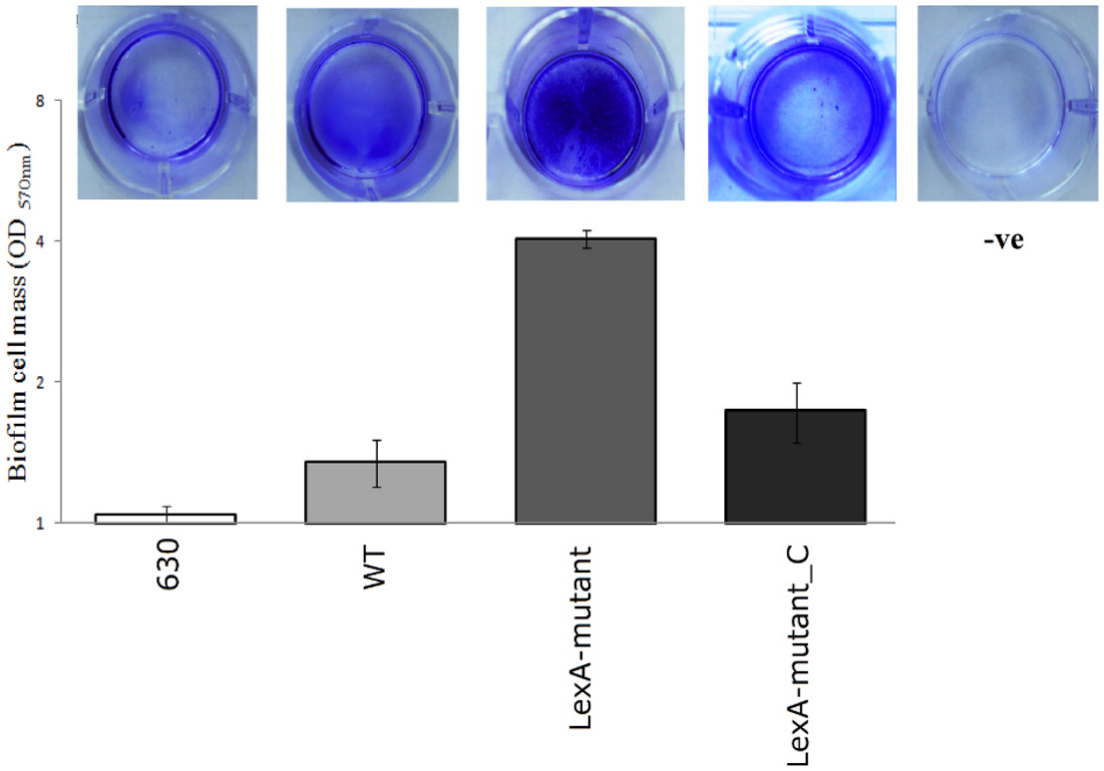
Biofilm formation after 24 h by *C*. *difficile* 630, R20291 wild-type, *lexA* mutant and complemented *lexA* mutant Top, pictures of biofilm after crystal violet staining corresponding to the bottom columns presenting numerical value of average OD_570_ readings from at least 3 independent experiments (reading taken in triplicates). Error bars shows standard deviation of the repeats. Top picture labelled as “–ve” shows the water only, negative control which was deducted from final readings before graphical presentation.

We assayed the *lexA* mutant strain for biofilm formation and compared the data to the characteristics of CD630 (control strain), R20291 (wild-type) and the complemented *lexA* mutant (Fig 6). The average optical density (OD_570_) measurements of biofilm cell mass for CD630, R20291, the *lexA* mutant and the complemented *lexA* mutant strains were OD_570_ = 1.0(±0.07); OD_570_ = 1.4(±0.26); OD_570_ = 4.1(±0.32); and OD_570_ = 1.7(±0.44), respectively (Fig 6). The R20291 wild-type strain produced more biofilm than the CD630 strain (Fig 6), which is in agreement with previous study [28]. The *C*. *difficile lexA* mutant strain produced significantly more biofilm than the R20291 wild type and CD630 strains. The complemented *lexA* mutant essentially restored the wild-type phenotype (Fig 6). In other species, SOS inductory antibiotics, such as quinolones and aminoglycosides, were shown to induce biofilm formation through the interplay between RecA and LexA [45]. Here we show, for the first time, that biofilm production in *C*. *difficile* can be stimulated by induction of the SOS response.

**Fig 7:**
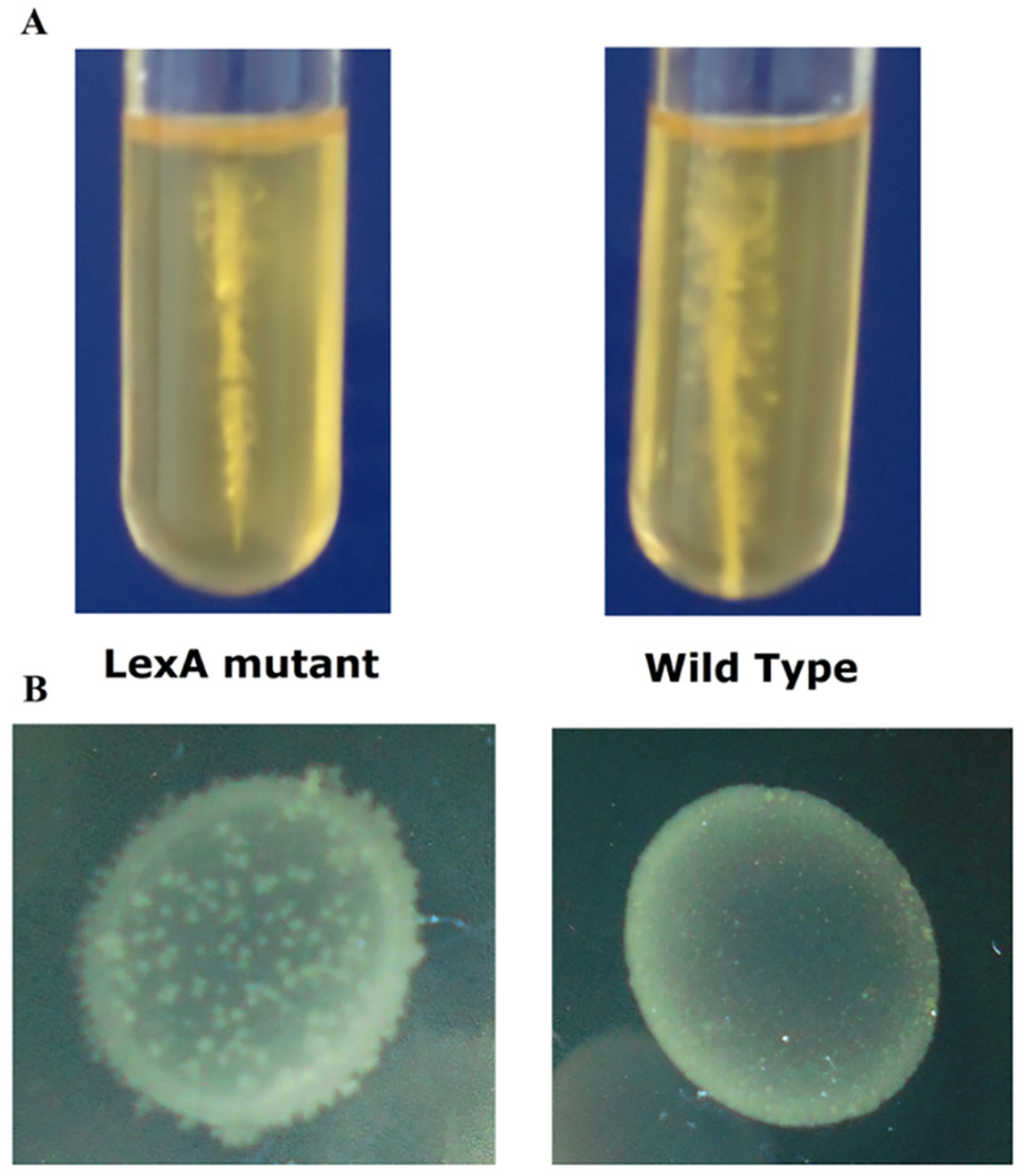
The motility of the *C*. *difficile lexA* mutant and its wild-type after 48 h. (A) Reduced swimming motility of the *lexA* mutant in 0.5% BHIS pre-reduced medium. (B) Increased gliding motility of the *lexA* mutant in 0.5% BHIS pre-reduced medium. The experiments were performed in two biological replicates, each in triplicate.

The possible effect of inactivated *lexA* on cell motility was also assessed. We tested the ability of the *lexA* mutant cells to swim, through stabbing of cells into a sloppy agar (0.5%, w/v), and the ability to glide, through inoculation onto the surface of agar. Fig 7A shows the results of the swimming motility assays. The wild-type (on the right) showed migration through the agar and the formation of multiple branches separated from the root. In contrast, the *lexA* mutant grew only within the hollow after stabbing. These data indicate that the *lexA* mutant is less able to swim than the wild-type. Similar observations were made in *P*. *aeruginosa*, where flagellum related motility was repressed when the SOS response was induced by ciprofloxacin [9].

In contrast, the *lexA* mutant showed some gliding motility (formation of rhizoid edge) after 48 h, while R20291 seemed non-motile within the same time period (Fig 7B). The formation of SOS-inducible biofilm in *P*. *aeruginosa* was related to motility as the initial event facilitating biofilm development [9]. The observed increase in biofilm formation associated with the *lexA* mutant, coupled with observed ability to undergo gliding, suggests a similar relationship in *C*. *difficile* as observed in *P*. *aeruginosa*. The mechanism of this regulation in *P*. *aeruginosa* remains unclear.

### Insertional mutation in *lexA* prevented inhibition of toxin A production by levofloxacin

**Fig 8:**
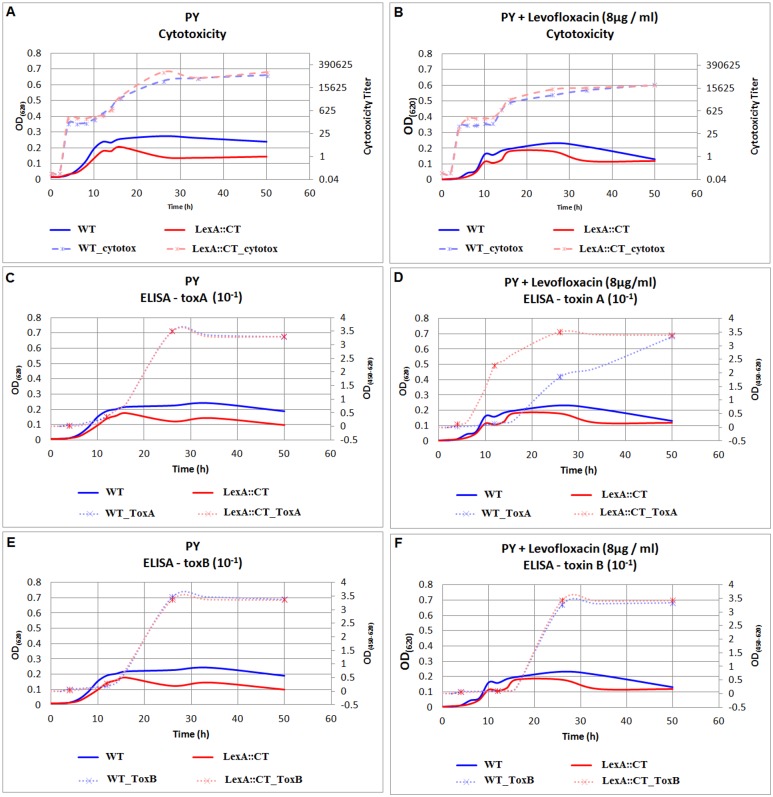
Toxin production by R20291 wild-type (WT) and the *lexA* mutant (LexA::CT) when grown in PY with and without levofloxacin supplementation. The line charts show the growth curve expressed as turbidity OD_620_ as a constant lines with the scale on the left; the cytotoxicity and ELISA toxicity expressed as cytotoxicity titre and ELISA OD_450-620_ as a broken lines with the scales on the right. The samples for cytotoxicity were taken as described for Fig 4 while the samples for ELISA were taken at 4, 12, 26 and 50 h which is shown as an asterisk on the growth curve.

In our previous work we showed *in vitro* LexA binding to the promoter region of the toxin A gene (*tcdA*) which is located within the pathogenicity locus PaLoc [12]. Here we followed toxins A and B production levels of the R20291 wild-type strain and its *lexA* mutant derivative by cytotoxicity assay and ELISA. The bacterial cells were grown in PY medium with and without 8 μg/ml LE to look for the suggested induction of the SOS response. Fig 8 shows the 50% cytotoxicity presented as a cytotoxicity titre of serial dilution (Fig 8A and 8B) as well as toxin specific ELISA (Fig 8C–8F) for the *C*. *difficile* R20291 parent strain and the *lexA* mutant (Standard deviation of replicas is shown in S3 File).

The cytotoxicity on Vero cells between the mutant and its isogenic parent when both grown in PY medium was comparable (Fig 8A). Cytotoxic activity was observed after only 4 h, in the early stages of exponential phase. This activity increased substantially after 16 h when the cells entered stationary phase. These data are broadly in agreement with the data of others [46]. The presence of LE in the growth media decreased cytotoxic activity produced by both strains by 1 log (Fig 8A and 8B). A similar effect has been previously reported [47, 48].

There was no difference in toxin B production, as measured by ELISA, either between the R20291 wild-type strain and the *lexA* mutant grown in PY medium (Fig 8E) or in PY supplemented with LE (8 μg/ml) (Fig 8F). Toxin B was first detected at the 26 h time point at comparable levels in both strains. In contrast, a two-fold decrease in the amount of toxin A produced was evident after 26 h (at 12 h: wt OD_(450–620)_ = 0, mutant OD_(450–620)_ = 2.5; at 26 h: wt OD_(450–620)_ = 1.75, mutant OD_(450–620)_ = 3.5) with the wild-type R20291 cells when LE was present (Fig 8C and 8D). The presence of LE had no measureable effect on the amount of toxin A produced by the *lexA* mutant (Fig 8C and 8D), but appeared to influence the timing of production. Thus, toxin A was detected at an earlier phase of growth when LE was present.

Different quinolone antibiotics have different effects on *C*. *difficile* toxin production. LE is known to decrease toxin production [47, 48] but also to induce the SOS response [34, 35]. In contrast, another SOS inducing fluoroquinolone, ciprofloxacin, increases *tcdA* expression and also shifts the initiation of expression into an earlier point in the growth phase [49]. Our data suggest that the inhibitory action of LE on toxin A production was countered by the constitutively induced SOS response present in the *lexA* mutant (Fig 8D). These data suggest that a fully induced SOS system is involved in the expression of the *tcdA* gene.

To date, the majority of studies support a model where the expression of the genes encoding both *C*. *difficile* toxins (A and B) are regulated simultaneously [50, 51]. The differences in toxin A and toxin B levels shown in this study, taken together with our previous finding that LexA binds to the *tcdA* promoter region [12] suggest, that the LexA regulated SOS response plays a role in the regulation of toxin A but not toxin B production.

### Increased sensitivity to metronidazole in *C*. *difficile* LexA mutant

Among the tested antibiotics (MIC determination in S4 Table): protein synthesis inhibitors (EM, TC, DC, CM), DNA/RNA synthesis inhibitors (LE, RI, MZ) and cell wall synthesis inhibitors (VA, PTc, AC, AM), dissimilarity was observed in the sensitivity to metronidazole and clindamycin (CM). The resistance of the *lexA* mutant to CM is due to the presence of the *ermB* located within the inserted ClosTron-derived intron which encodes for resistance to (LM) lincomycin (CM is LM derivate). The MIC_mz_ of the R20291 wild-type strain was determined as 1.5–2.0 μg/ml, while the MIC_mz_ of the *lexA* mutant was 0.5 μg/ml (S4 Table). The metronidazole susceptibility of *C*. *difficile* varies. Resistance has been shown to be heterogeneous and inducible but the precise mechanism has yet to be established [14, 52–56]. The observed increase in sensitivity to metronidazole in the *lexA* mutant is in contrast to a previous study describing increased expression of core SOS genes in a metronidazole resistant strain, CD26A54_R, relatively to its sensitive counterpart CD26A54_S [14]. The resistance mechanisms described in *Bacteroides fragilis* and *Helicobacter pylori* also include overexpression of *recA* and a DNA repair/recombination protein. The data presented here supports the notion that the mechanism of metronidazole resistance in *C*. *difficile* is connected to the SOS network. However, it also suggests that an increased SOS response is associated with an increase in metronidazole resistance rather than decreased sensitivity.

## Conclusions

The SOS response is widespread, being found in almost all eubacterial groups [1], but it varies significantly from species to species. Often, in addition to DNA repair, LexA controls various other functions, such as the expression of toxin genes [7] and antibiotic resistance determinants [57] or the transfer of genetic elements [6, 58].

In the present study we have generated the first LexA deficient mutant of *C*. *difficile* and provided experimental evidence that the *C*. *difficile* SOS response is based on the LexA-RecA* paradigm. Moreover, we demonstrated that in *C*. *difficile*, LexA is not only a regulator of the DNA damage response but also controls other biological functions related to virulence, notably motility, biofilm production, sporulation, toxin A production and metronidazole resistance. Our data imply that LE induces *recA* expression in *C*. *difficile* in a LexA-independent manner.

Our findings suggest that drugs which interfere with induction of the SOS response may not only provide next generation *C*. *difficile* antibiotics, as LexA seems to control several house-keeping genes [12], but also decrease its virulence potential.

## Supporting Information

S1 File Intron targeting and design tool results. (DOCX)

S2 File Standard deviation of R20291 wild-type and *lexA* mutant growth curves in various media. (PDF)

S3 File Standard deviation of R20291 wild-type and *lexA* mutant cytotoxicity and ELISA. (PDF)

S1 Table List of strains and plasmids used in this study. (DOCX)

S2 Table List of oligonucleotides used in this study. (XLSX)

S3 Table The calculation of *recA* fold difference in expression. (DOCX)

S4 Table Antimicrobial susceptibility testing of *C*. *difficile* R20291 wt and R20291-LexA::CT mutant. (DOCX)
