# Supplementary material for: The SOS Response Master Regulator LexA Is Associated with Sporulation, Motility and Biofilm Formation in Clostridium difficile
Source: PLoS One. 2015 Dec 18;10(12):e0144763. doi: 10.1371/journal.pone.0144763 (PMC4689574; doi:10.1371/journal.pone.0144763)
Supplement: S1 File — (DOCX) [file pone.0144763.s001.docx]

S1 File. Intron targeting and design tool results.

=====================================================

An intron targeting and design tool is available at

http://clostron.com/ free of charge (Heap et al., 2010).

On 24-October-2012 possible intron target sites were identified in

the sequence of interest "C.diff_LexA" using the Perutka method.

Query sequence: C.diff_LexA

--------------

ATGAGTGATGTTATGTATTTAGATCTAACTGAAAAGCAAGTCTTGATATTGGAATTTATAAAGTC

TCAAATCATATTAAAAGGTTATCCACCTGCTGTAAGAGAAATATGTACTGCTGTAGGGTTAAGAT

CTACTTCAACTGTGCACTCTCATCTAAATAAACTTGAAAAACTGGGATACATAAGAAAGGACCCT

ACTAAACCAAGAGCTATTGAAGTTTTAGAACGTAGTAAAGTAAATGATGTTTCTGGAGCTAATCA

AGAAATAATAGAGCTTCCTTTAGTAGGTCAAATAACAGCTGGAGAACCCATTTTAGCTCAACAAA

ACATAGAGGAATACATTCCATTCCCTGCCAGTTTAGTAAAGGGCAGTAACAATTTTGTATTAAGA

GTAAAAGGCGAAAGTATGATTAATGCAGGAATTTTAGATGAAGATTACGTTGTAGTAGACAAAAA

AAATACAGCCTTAAATTCTCAGATAGTAGTTGCACTTATAAATGGTGAATCTGCTACAGTTAAGA

GGTTTTTTAAAGAAGGAAATTTAATAAGACTTCAGCCAGAAAATGATTTTATGGAACCAATTATG

CTCAATGACTCAGAAGTTGAAATTGTAGGTATTGTAACTGGTGTATTTAGAGTTATTAAATAG

Results of target site identification algorithm

-----------------------------------------------

Intron

_||_

\ /

\/

Sequence Exon--><--Exon Pos Score

GTTGAAGTAGATCTTAACCCTACAGCAGTACATATTTCTCTTACA 110|111a 10.746

ATGCTCAATGACTCAGAAGTTGAAATTGTAGGTATTGTAACTGGT 612|613s 6.985

CATTTTCTGGCTGAAGTCTTATTAAATTTCCTTCTTTAAAAAACC 535|536a 6.156

GGAATTTTAGATGAAGATTACGTTGTAGTAGACAAAAAAAATACA 447|448s 5.954

TCAGAAGTTGAAATTGTAGGTATTGTAACTGGTGTATTTAGAGTT 624|625s 5.471

CTTCAGCCAGAAAATGATTTTATGGAACCAATTATGCTCAATGAC 579|580s 5.168

**TTATTTCTTGATTAGCTCCAGAAACATCATTTACTTTACTACGTT 238|239a 5.137**

TGAGTCATTGAGCATAATTGGTTCCATAAAATCATTTTCTGGCTG 567|568a 4.997

CCCATTTTAGCTCAACAAAACATAGAGGAATACATTCCATTCCCT 336|337s 4.786

TCTGGAGCTAATCAAGAAATAATAGAGCTTCCTTTAGTAGGTCAA 276|277s 4.772

AAGGACCCTACTAAACCAAGAGCTATTGAAGTTTTAGAACGTAGT 216|217s 4.595

GATCTAACTGAAAAGCAAGTCTTGATATTGGAATTTATAAAGTCT 51|52s 4.133

GTAGATCTTAACCCTACAGCAGTACATATTTCTCTTACAGCAGGT 104|105a 4.001

ATGTATTTAGATCTAACTGAAAAGCAAGTCTTGATATTGGAATTT 42|43s 3.733

GATTAGCTCCAGAAACATCATTTACTTTACTACGTTCTAAAACTT 229|230a 3.717

TGCACAGTTGAAGTAGATCTTAACCCTACAGCAGTACATATTTCT 116|117a 3.578

TGTTTTGTTGAGCTAAAATGGGTTCTCCAGCTGTTATTTGACCTA 298|299a 3.566

AAAGTAAATGATGTTTCTGGAGCTAATCAAGAAATAATAGAGCTT 261|262s 3.485

TTTTGTTGAGCTAAAATGGGTTCTCCAGCTGTTATTTGACCTACT 296|297a 3.396

GCAAGTCTTGATATTGGAATTTATAAAGTCTCAAATCATATTAAA 65|66s 2.928

ACAGCCTTAAATTCTCAGATAGTAGTTGCACTTATAAATGGTGAA 489|490s 2.843

CTGTTATTTGACCTACTAAAGGAAGCTCTATTATTTCTTGATTAG 268|269a 2.754

TCTCATCTAAATAAACTTGAAAAACTGGGATACATAAGAAAGGAC 177|178s 2.728

ACTTCAATAGCTCTTGGTTTAGTAGGGTCCTTTCTTATGTATCCC 188|189a 2.698

GTAAATGATGTTTCTGGAGCTAATCAAGAAATAATAGAGCTTCCT 264|265s 2.690

Selected target site

--------------------

Name (position): 238a

Sequence: TTATTTCTTGATTAGCTCCAGAAACATCATTTACTTTACTACGTT

Primers for conventional SOE PCR intron re-targeting

----------------------------------------------------

C.diff_LexA-238a-IBS primer: AAAAAAGCTTATAATTATCCTTACAGAACCATCATGTGCGCCCAGATAGGGTG

C.diff_LexA-238a-EBS1d primer: CAGATTGTACAAATGTGGTGATAACAGATAAGTCCATCATTTTAACTTACCTTTCTTTGT

C.diff_LexA-238a-EBS2 primer: TGAACGCAAGTTTCTAATTTCGGTTTTCTGTCGATAGAGGAAAGTGTCT

EBS universal primer: CGAAATTAGAAACTTGCGTTCAGTAAAC

Intron targeting region: C.diff_LexA-238a

-----------------------

AAGCTTATAATTATCCTTACAGAACCATCATGTGCGCCCAGATAGGGTGTTAAGTCAAGTAGTTT

AAGGTACTACTCTGTAAGATAACACAGAAAACAGCCAACCTAACCGAAAAGCGAAAGCTGATACG

GGAACAGAGCACGGTTGGAAAGCGATGAGTTACCTAAAGACAATCGGGTACGACTGAGTCGCAAT

GTTAATCAGATATAAGGTATAAGTTGTGTTTACTGAACGCAAGTTTCTAATTTCGGTTTTCTGTC

GATAGAGGAAAGTGTCTGAAACCTCTAGTACAAAGAAAGGTAAGTTAAAATGATGGACTTATCTG

TTATCACCACATTTGTACA
