# Supplementary material for: The SOS Response Master Regulator LexA Is Associated with Sporulation, Motility and Biofilm Formation in Clostridium difficile
Source: PLoS One. 2015 Dec 18;10(12):e0144763. doi: 10.1371/journal.pone.0144763 (PMC4689574; doi:10.1371/journal.pone.0144763)

S2 File. Standard deviation of R20291 wild-type and *lexA* mutant growth curves in various media

BHIS

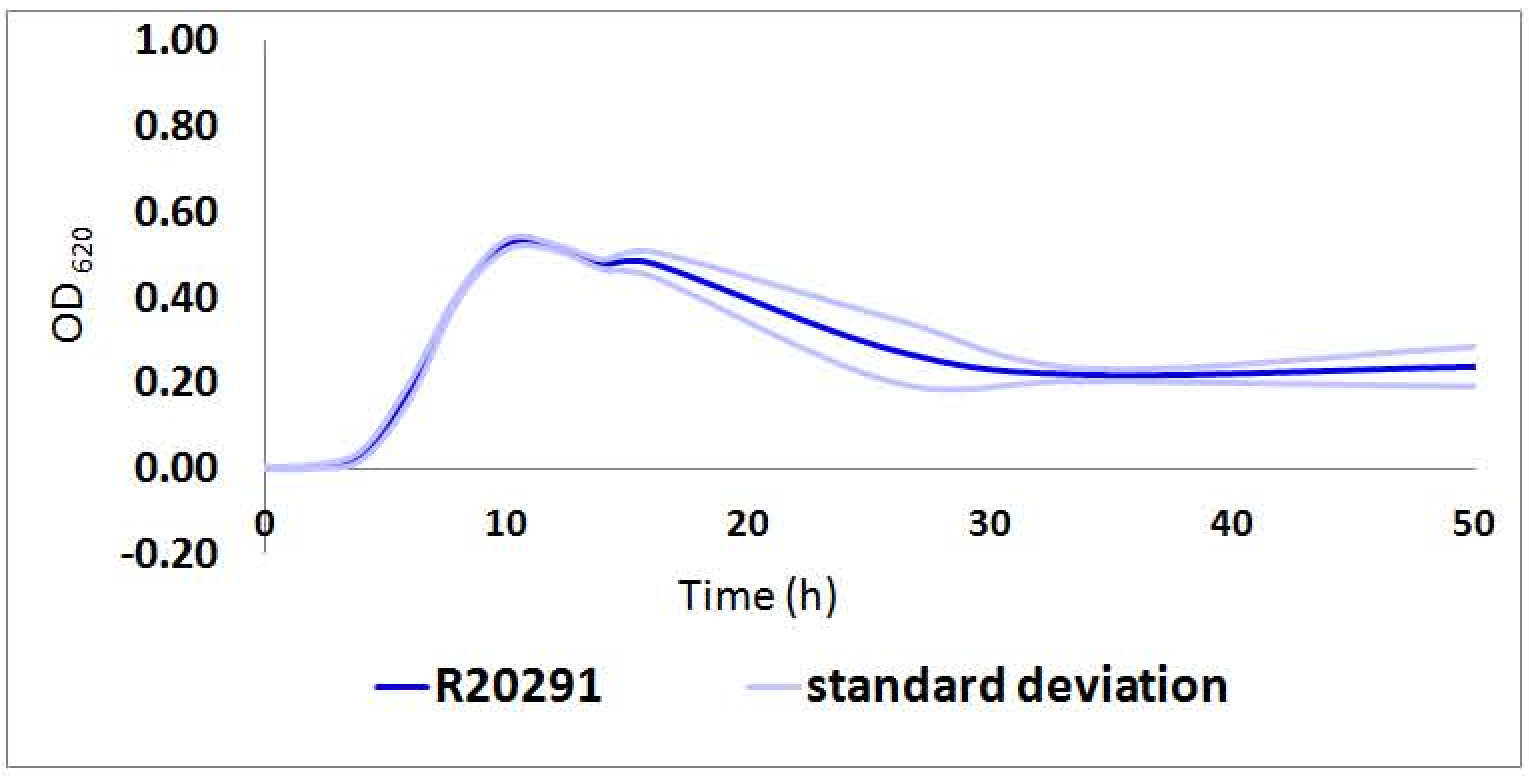

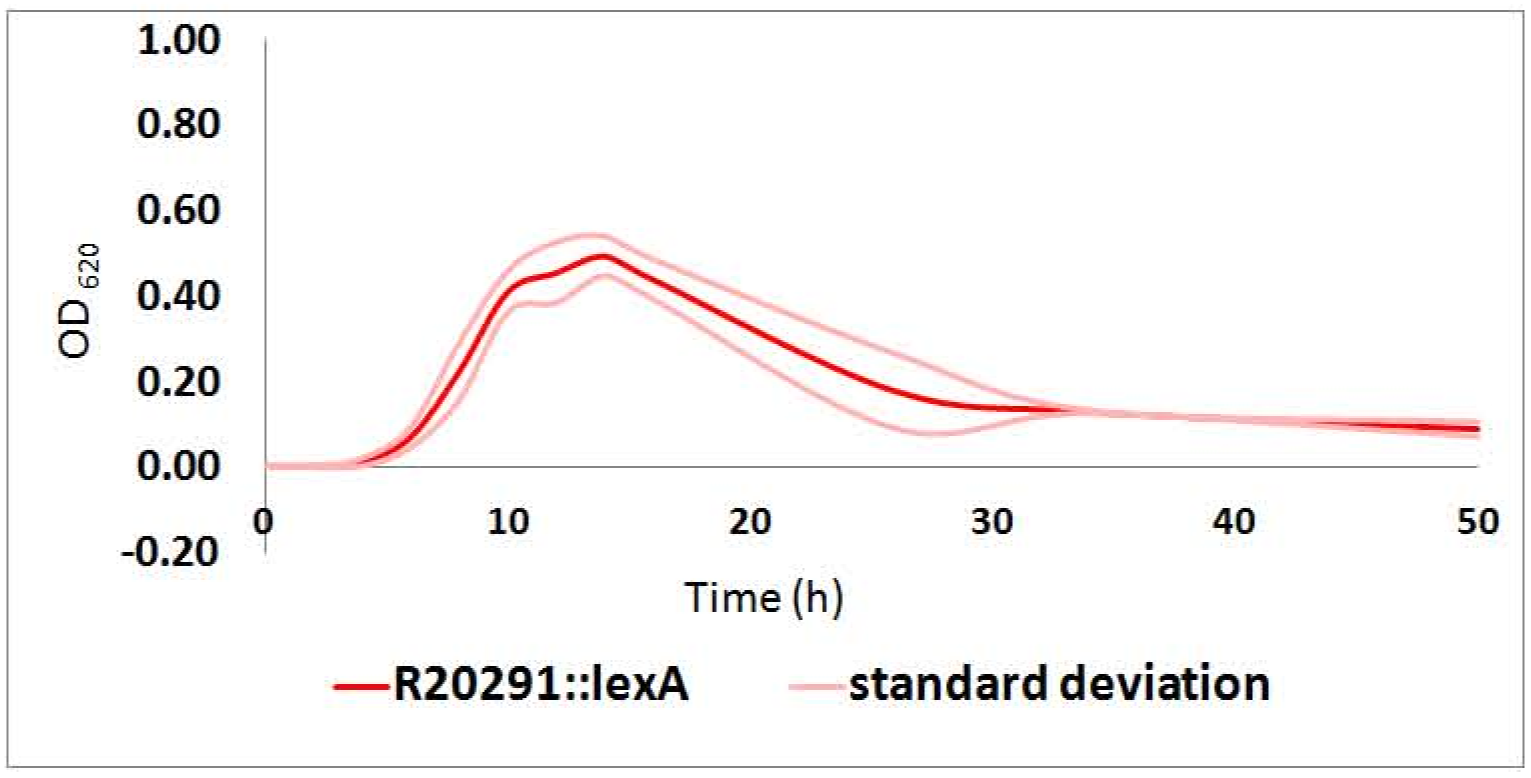

BHISG

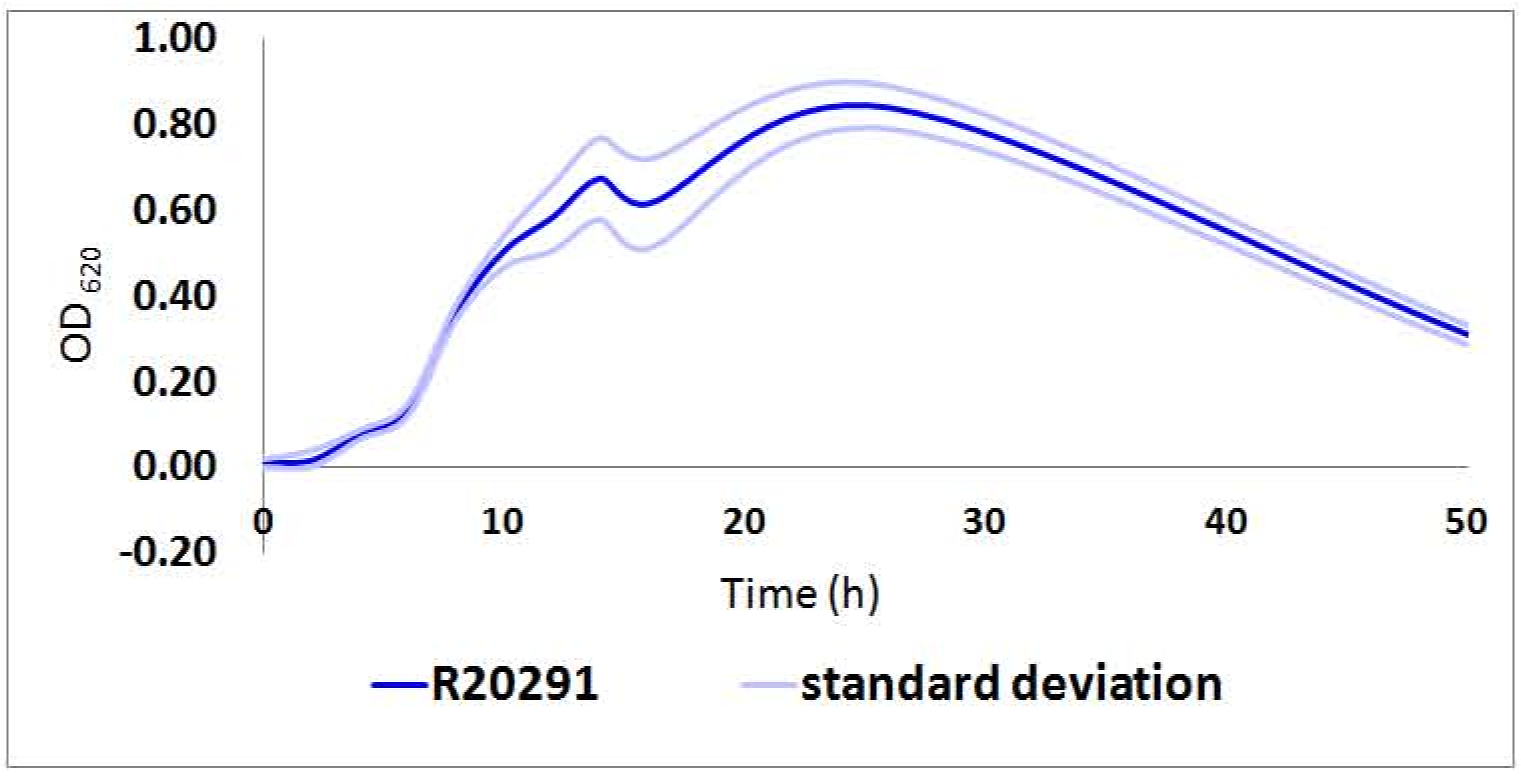

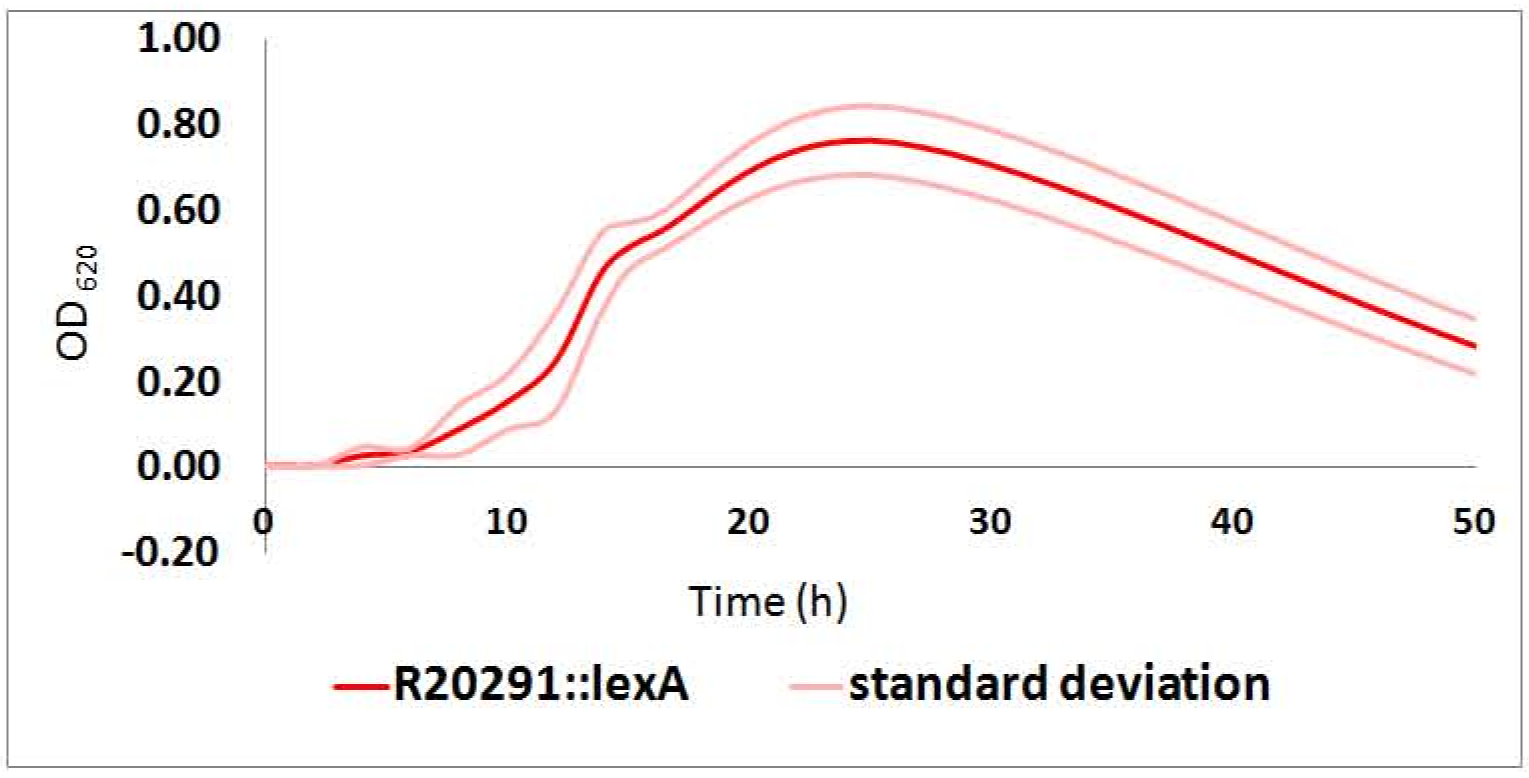

PY

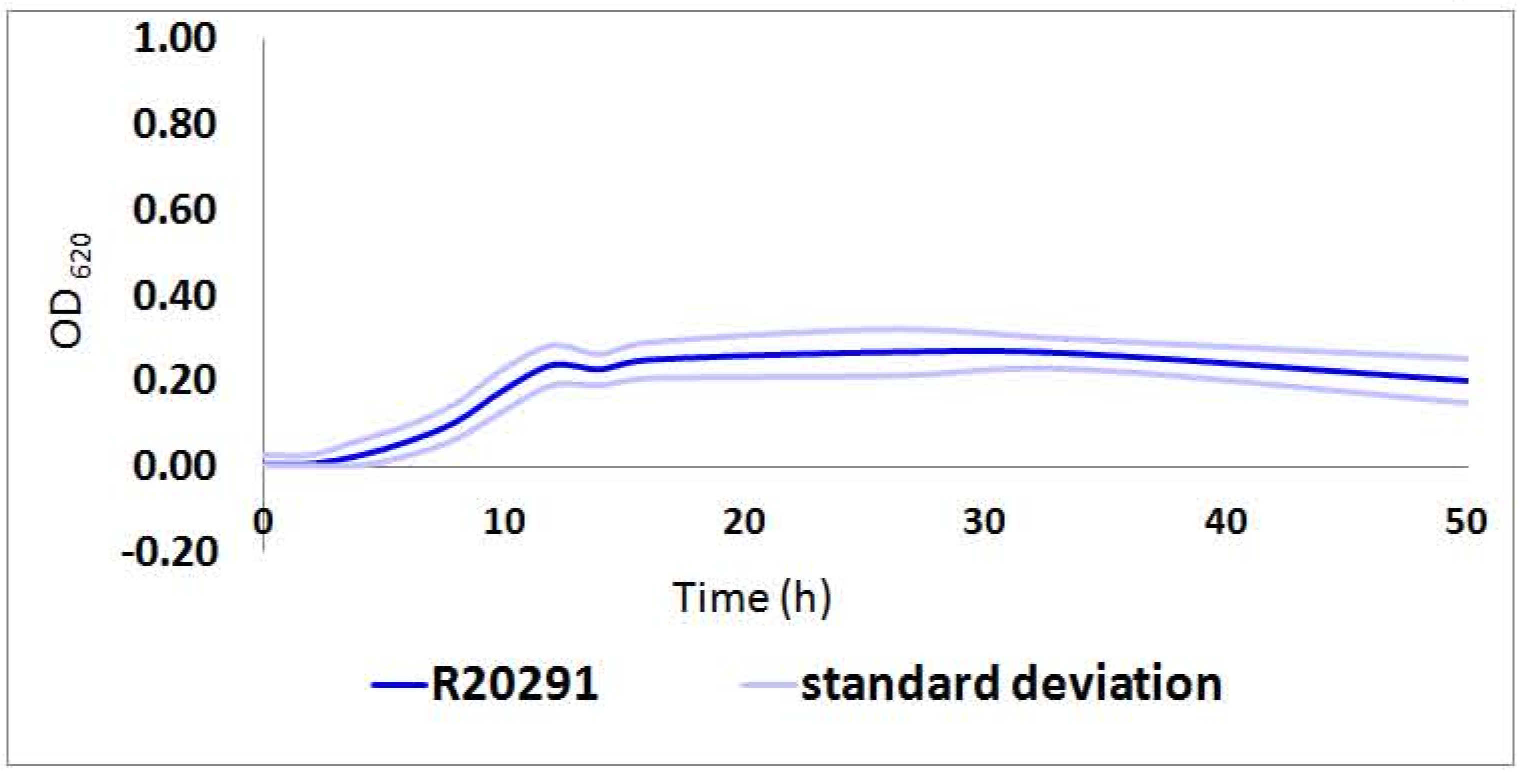

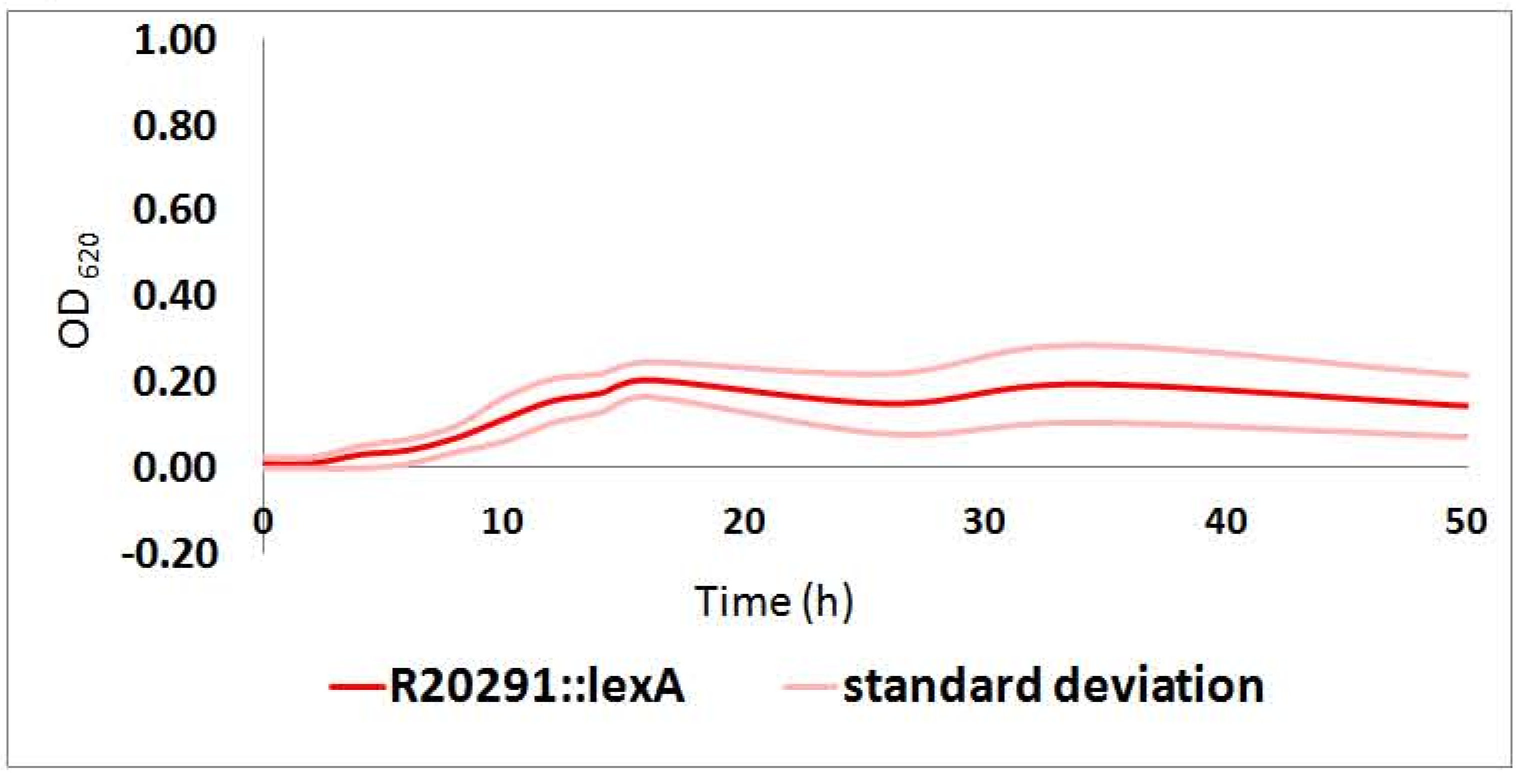

PYG

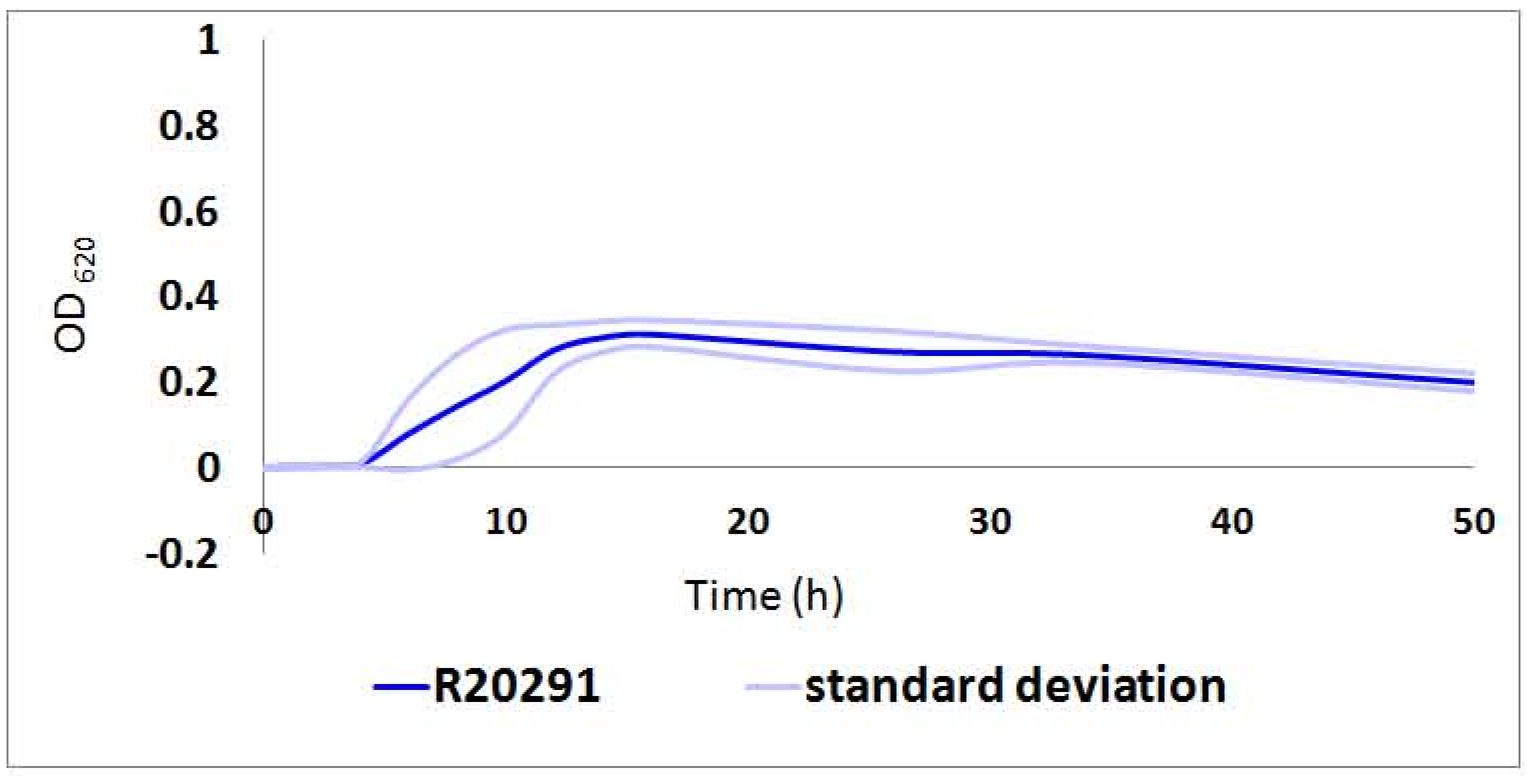

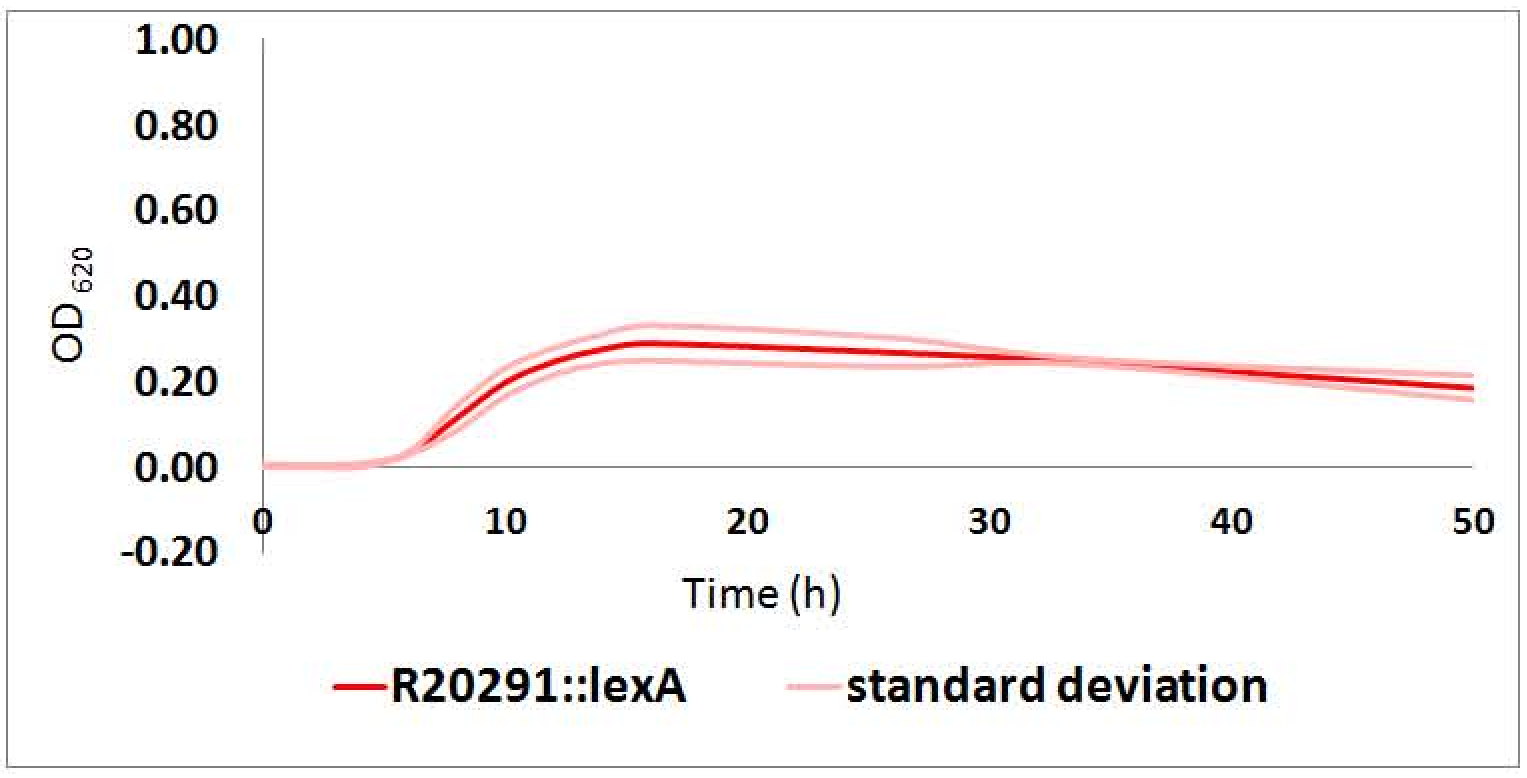

PY + Levofloxacin (8µg/ml)

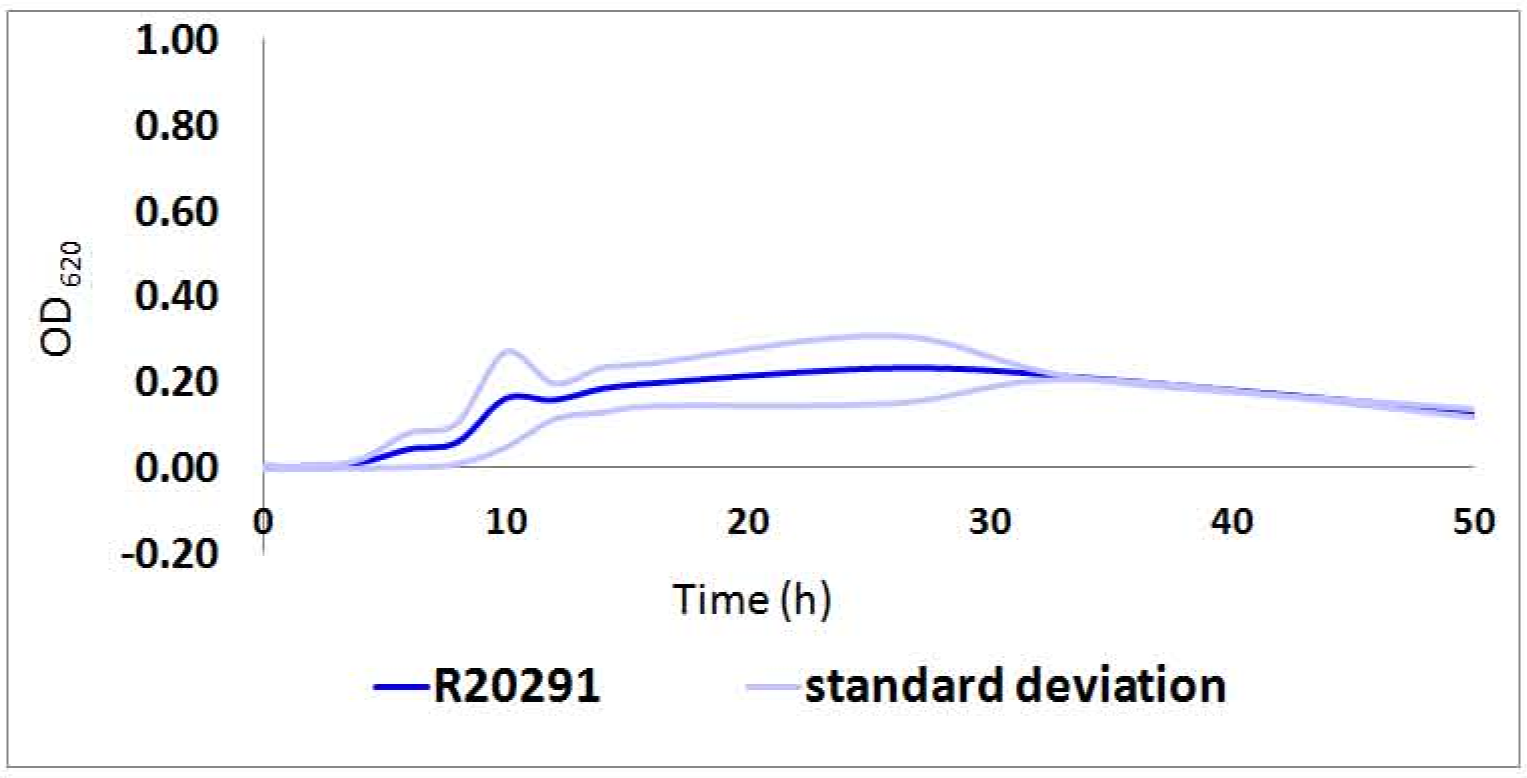

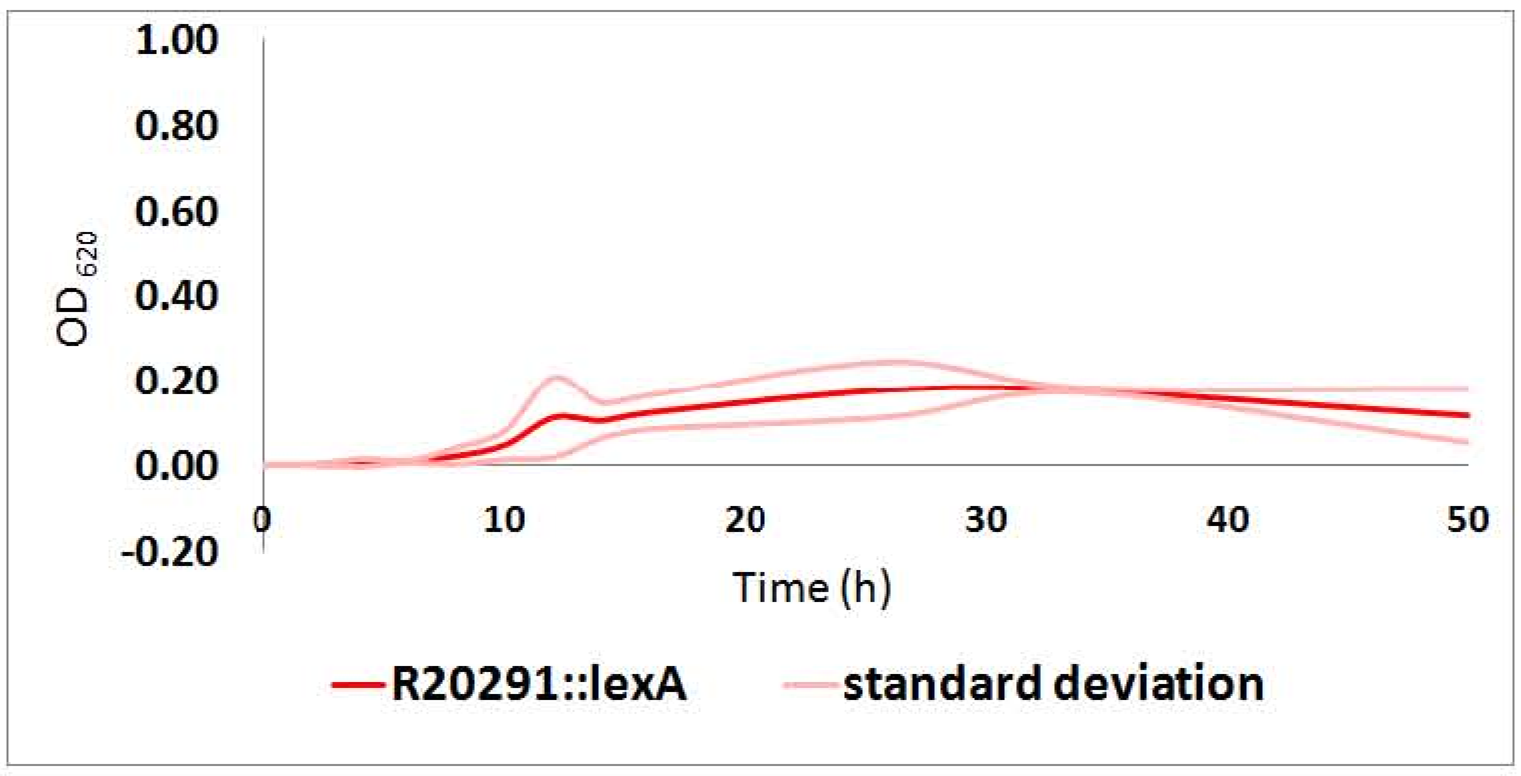

Supplement: S2 File — (PDF) [file pone.0144763.s002.pdf]
