# Supplementary figures and images for: The SOS Response Master Regulator LexA Is Associated with Sporulation, Motility and Biofilm Formation in Clostridium difficile
Source: PLoS One. 2015 Dec 18;10(12):e0144763. doi: 10.1371/journal.pone.0144763 (PMC4689574; doi:10.1371/journal.pone.0144763)

S3 File. Standard deviations of R20291 wild type and *lexA* mutant

CYTOTOXICITY AND ELISA

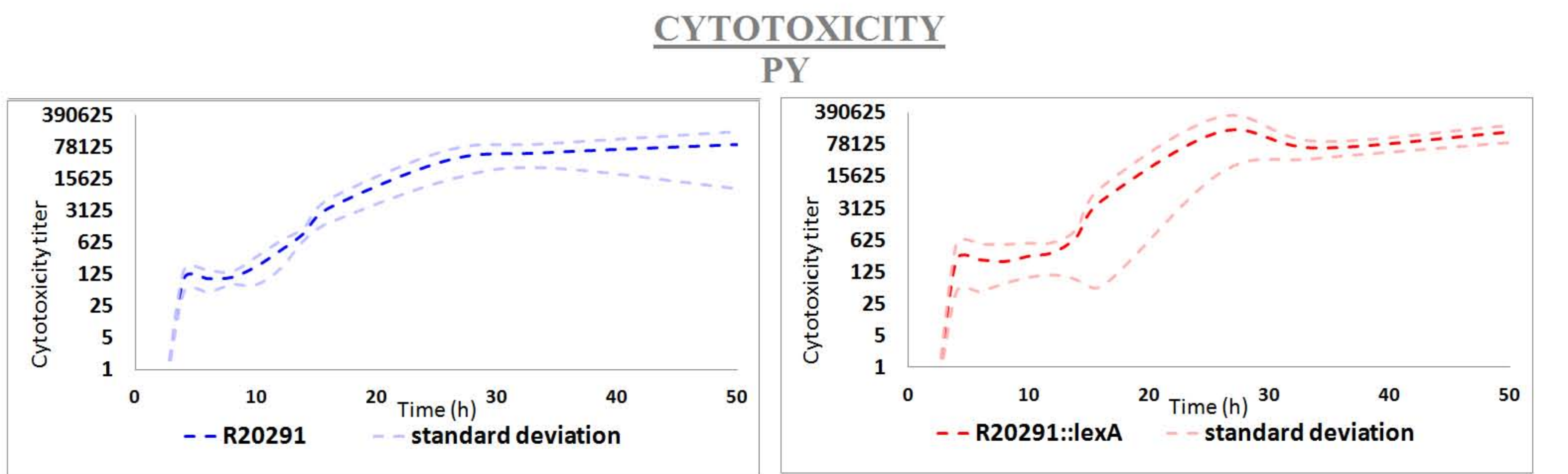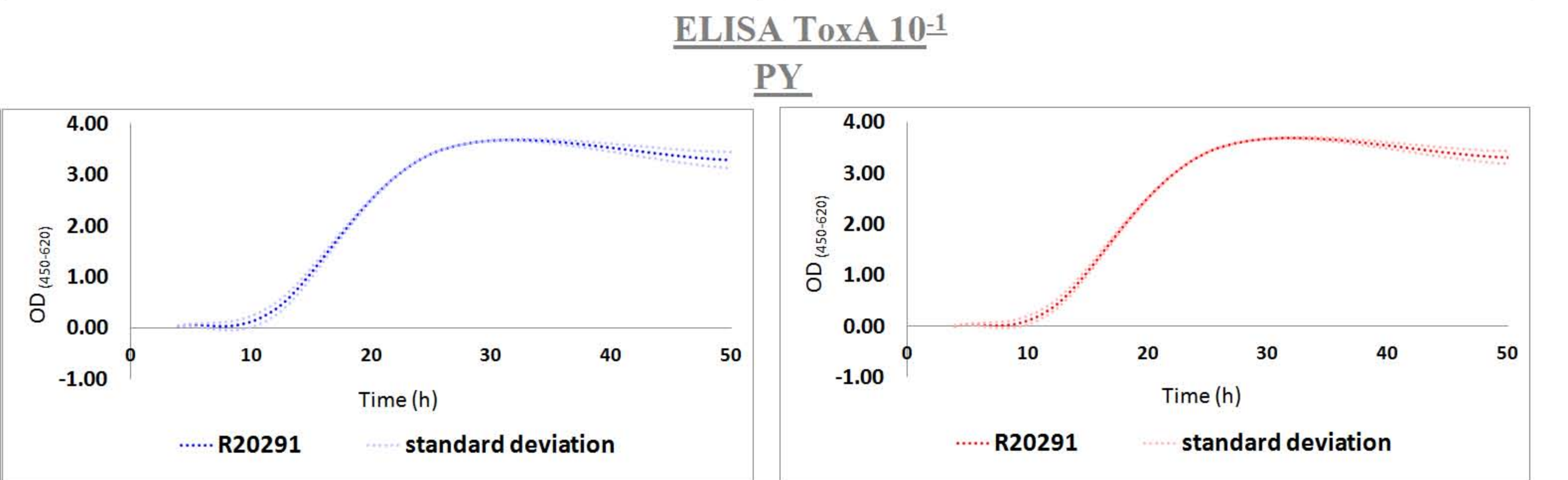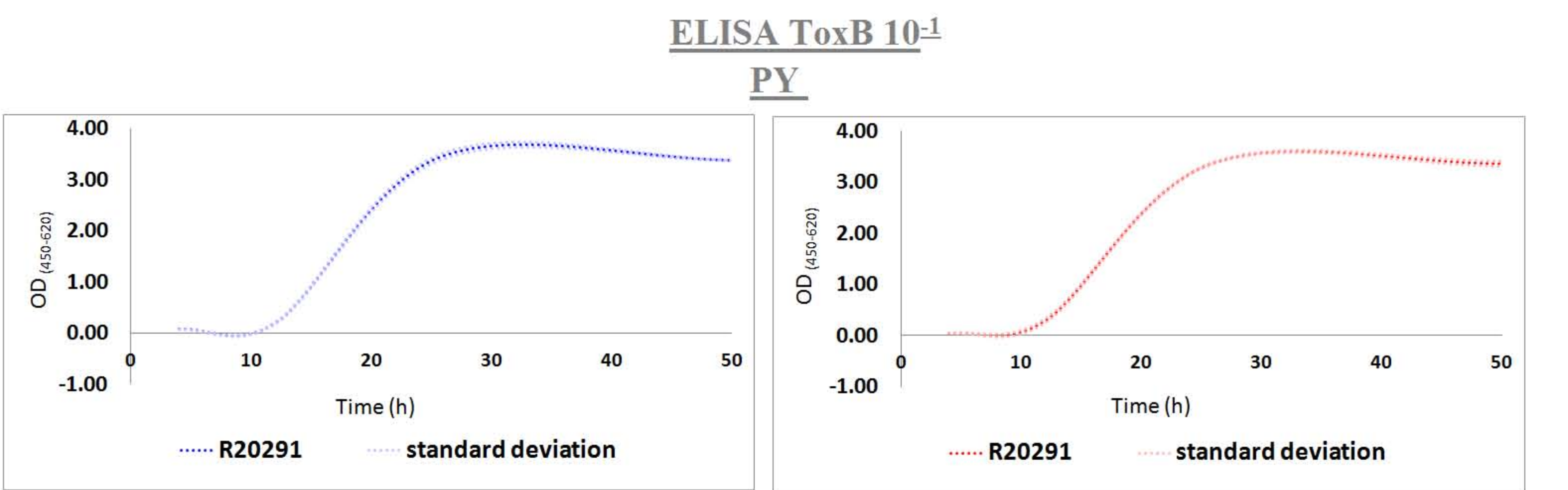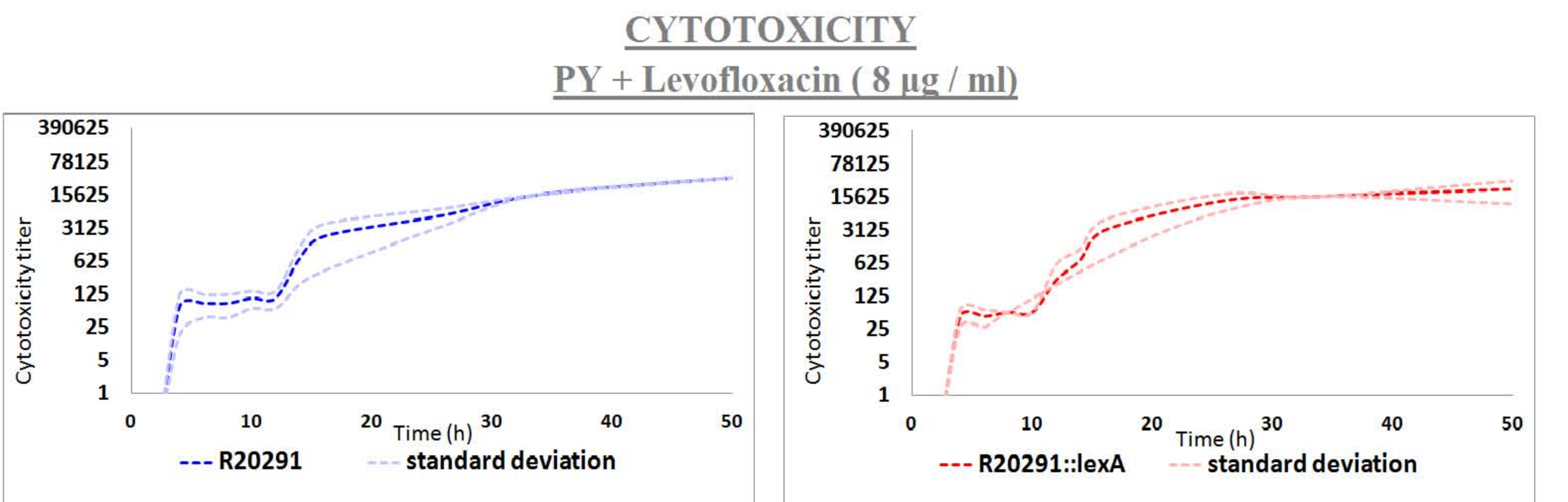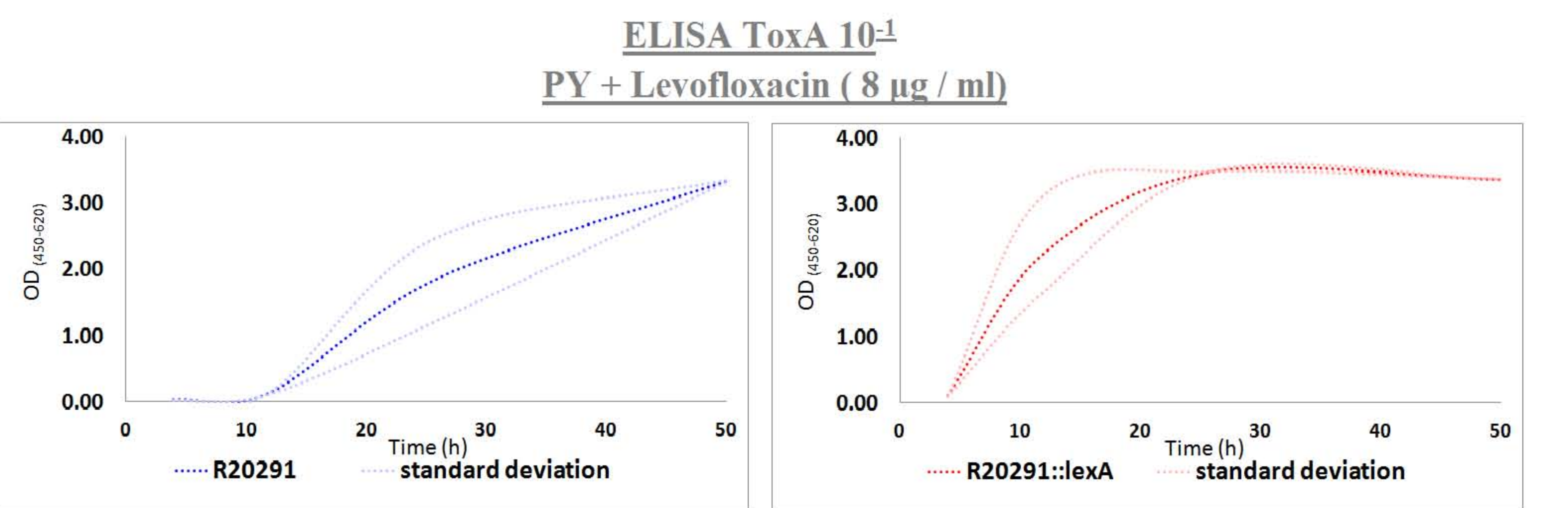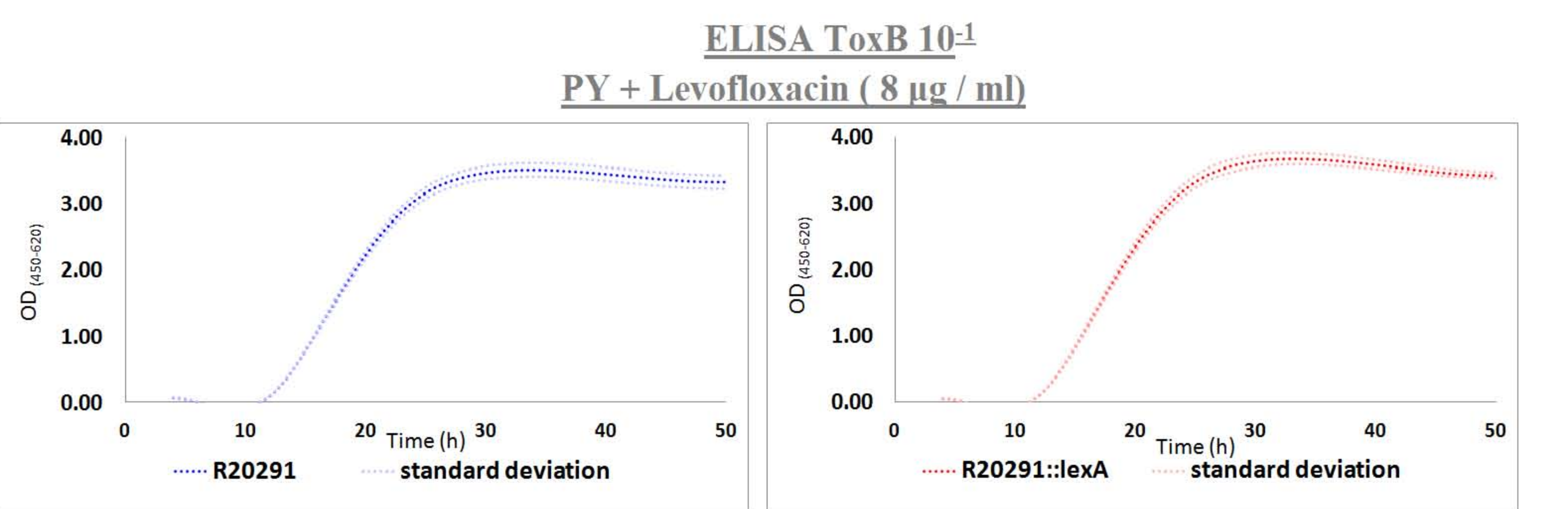

Supplement: S3 File — (PDF) [file pone.0144763.s003.pdf]
