# Supplementary material for: The SOS Response Master Regulator LexA Is Associated with Sporulation, Motility and Biofilm Formation in Clostridium difficile
Source: PLoS One. 2015 Dec 18;10(12):e0144763. doi: 10.1371/journal.pone.0144763 (PMC4689574; doi:10.1371/journal.pone.0144763)
Supplement: S1 Table — (DOCX) [file pone.0144763.s004.docx]

S1_Table. List of strains and plasmids used in the study

| **Strain** | **Strain information** | **Source/reference** |
| --- | --- | --- |
| *Escherichia coli* TOP 10 | Cloning host | Invitrogen (CRG, University of Nottingham culture collection) |
| *Escherichia coli* CA434 | Conjugation donor | CRG, University of Nottingham culture collection |
| *Clostridium difficile* 630 | PCR-ribotype 012 | ZZV culture collection |
| *Clostridium difficile* R20291 | BI/NAP1/027 (Stoke Mandeville, UK, Anaerobe Reference Laboratory, Cardiff | CRG, University of Nottingham culture collection |
| *CDiR20291-lexA238a::CT* | *C. difficile* R20291 with ClosTron mutation in *lexA* gene | This study |
| pMTL007C-E2::LexA238a | ClosTron plasmid carrying re-targeted site for insertional knock out of *lexA* gene at 238::239 antisense site | This study |
| pMTL84151::lexA | complementation plasmid carrying *lexA* gene (gene bank accession no: FJ797649.1) | This study |
