# Supplementary material for: The SOS Response Master Regulator LexA Is Associated with Sporulation, Motility and Biofilm Formation in Clostridium difficile
Source: PLoS One. 2015 Dec 18;10(12):e0144763. doi: 10.1371/journal.pone.0144763 (PMC4689574; doi:10.1371/journal.pone.0144763)
Supplement: S3 Table — (DOCX) [file pone.0144763.s006.docx]

S3 Table. The calculation of *recA* fold difference in expression.

| **Sample no** | **Sample name** | **time point** | **test sample average ∆Ct** | **Calibrator (R20291) average ∆Ct** | **∆∆CT(∆CT test - ∆CT calibrator)** | **Fold difference in test sample relative to calibrator** |
| --- | --- | --- | --- | --- | --- | --- |
| **1** | *lexA* mutant | 12 h | -1.845±0.34 | -1.2 | -0.645 | **1.56**  (1.23-1.98) |
|  |  | 24 h | -1.035±0.03 | 0.63 | -1.665 | **3.17**  (3.10-3.23) |
| **2** | R20291 wild type + Levofloxacin | 12 h | -2.5±1.1 | -1.2 | -1.3 | **2.46**  (1.15 - 5.27) |
|  |  | 24 h | -2.16±0.84 | 0.63 | -2.79 | **6.91**  (3.86 - 12.38) |
| **3** | *lexA* mutant + Levofloxacin | 12 h | -2.52±1.25 | -1.2 | -1.32 | **2.49**  (1.04-5.94) |
|  |  | 24 h | -5.57±1.42 | 0.63 | -6.2 | **73.52**  (27.47-196.72) |

The ∆Ct refers to the difference in Ct value of *recA* and the normalisation gene *rpsJ*. The average ∆Ct of the test sample and the calibrator were calculated on the basis of at least 4 independent values (based on biological and technical replicates.) The last column shows the fold difference in test sample relative to calibrator calculated with the equation $2^{{-1\Delta\Delta C}_{T}}$.
