# Supplementary material for: The SOS Response Master Regulator LexA Is Associated with Sporulation, Motility and Biofilm Formation in Clostridium difficile
Source: PLoS One. 2015 Dec 18;10(12):e0144763. doi: 10.1371/journal.pone.0144763 (PMC4689574; doi:10.1371/journal.pone.0144763)
Supplement: S4 Table — (DOCX) [file pone.0144763.s007.docx]

S4 Table. Antimicrobial susceptibility testing of the *C. difficile* R20291 wt and R20291-LexA::CT mutant

| **Class** | **Symbol** | **Antibiotic** | **AST method** | **Antibiotic concentration**  **(µg/ml)** | **MIC breakpoint** | **Medium** | **MIC**  **(µg/ml)** | **MIC**  **(µg/ml)** |
| --- | --- | --- | --- | --- | --- | --- | --- | --- |
|  |  |  |  |  |  |  | **R20291 wt** | **R20291-LexA::CT** |
| Protein  synthesis  inhibitors | EM | Erythromycin | Etest® | 0.016-256 | 8^b^ | COS* | >256 | >256 |
|  | TC | Tetracycline | Etest® | 0.016-256 | 8^b^ | COS | 0,19 | 0,125 |
|  | DC | Doxycycline | Etest® | 0.016-256 | ND | COS | 0.032 | 0,047 |
|  | CM | Clindamycin  (LM derivate) | Etest® | 0.016-256 | 8^b^ | COS | 1.5 | >256 |
| DNA/RNA  synthesis  inhibitors |  |  |  |  |  |  |  |  |
|  | LE | Levofloxacin | Etest® | 0.002-32 | 4-8^d^ | COS | >32 | >32 |
|  | RI | Rifampicin | Etest® | 0.002-32 | 0.004^a^ | COS | 0.003 | 0,003 |
|  | **MZ** | **Metronidazole** | Etest® | **0.016-256** | **2^a^** | **COS** | **1.5-2.0** | **0.5** |
|  |  |  |  |  |  |  |  |  |
| Cell wall  synthesis  inhibitors | VA | Vancomycin | Etest® | 0.016-256 | 2^a^ | COS | 0.5 | 0.75 |
|  | PTc | Pipera/Tazo | Etest® | 0.016-256 | ND | COS | 12 | 8 |
|  | AC | Amoxicillin | Etest® | 0.016-256 | 16^c^ | COS | 1.0 | 0,75 |
|  | AM | Ampicillin | AD | 0.125-32 | ND | BHIS | 8 | 4 |
|  | AM | Ampicillin | AD | 0.125-32 | ND | PY | 4 | 4 |

*COS plates, BHIS broth, PY broth

AD – agar dilution

ND not defined by EUCAST

^a^ ([EUCAST 2015](#_ENREF_1))

^b^ ([Lachowicz D. *et al.,* 2015](#_ENREF_3))

^c^ ([Keessen E. C. *et al.,* 2013](#_ENREF_2))

^d^ ([Spigaglia P. *et al.,* 2008](#_ENREF_4))
